# Changes of Microbiome in Human Papillomavirus Infection and Cervical Cancer: A Systematic Review and Meta‐Analysis

**DOI:** 10.1002/cnr2.70246

**Published:** 2025-06-02

**Authors:** Wei Zhang, Yan Ge, Lihe Yao, Qingchun Yan, Jiuju Wei, Yanfei Yin, Bin Liu

**Affiliations:** ^1^ The First School of Clinical Medicine Lanzhou University Lanzhou China; ^2^ The School of Stomatology Lanzhou University Lanzhou China; ^3^ Healthy Examination & Management Center of Lanzhou University Second Hospital Lanzhou China; ^4^ Department of Gynecology Lanzhou University First Hospital Lanzhou China

**Keywords:** cervical cancer, high‐throughput sequencing, HPV virus, meta‐analysis, microbiota

## Abstract

**Background and Objectives:**

We aimed to conduct a systematic review and meta‐analysis of high‐throughput sequencing studies to assess changes in microbiome alpha, beta diversity, and composition differences in patients with human papillomavirus (HPV) infection and cervical cancer.

**Methods:**

The PubMed, Embase, Web of Science, and Cochrane Library databases were systematically searched to include original studies. The effect size estimates with a 95% confidence interval were combined using a random effects model. The meta‐analysis was performed using the Stata MP16 software.

**Results:**

A total of 64 studies were included, with a meta‐analysis of the diversity index performed on a subset of seven studies. Microbial diversity of patients infected with HPV was observed to be significantly different from that of healthy controls (CHAO index: 95% CI 0.42, 5.03, *I* ^2^ = 99.18%, *p* < 0.05). Subgroup analysis based on the sample collection region showed a significant difference between vaginal microbiota of the treatment group and control group, as measured by the Shannon index (95% CI 0.12, 0.97, *I* ^2^ = 67.09%, *p* < 0.05). Further, subgroup analysis of samples sequenced with the primer pair for the V3–V4 region showed a statistically significant difference in alpha diversity (Shannon index: 95% CI 0.28, 0.72, *I* ^2^ = 0.00%, *p* < 0.05) between treatment and control groups. The microbial diversity varied between patients with inferior cervical lesions (low‐grade squamous intraepithelial lesion) and healthy controls (Shannon index: 95% CI 0.02, 0.58, *I* ^2^ = 0.00%, *p* < 0.05). The bacterial marker genera differed at each cervical lesion stage. *Gardnerella* was prevalent during the HPV infection stage, but its proportion decreased after the occurrence of cervical lesions. In contrast, the proportions of *Prevotella*, *Porphyromonas*, and *Dialister* increased during the cervical cancer stages.

**Conclusions:**

Patients with simple HPV infections frequently exhibit unstable microbial diversity and are influenced by various factors. The microbial environment continues to change after the occurrence of cervical lesions and is correlated with the severity of cervical lesions.

## 1 Introduction

Cervical cancer (CC) is the fourth most common cancer threatening women's health worldwide, with approximately 600,000 cases and 300,000 deaths annually [1]. Over 99% of precancerous lesions are associated with human papillomavirus (HPV) infection, with high‐risk HPV types 16, 18, 31, 33, and 45, which are identified in approximately 97% of CC cases [2]. Recently, many studies have found that an imbalance in the cervicovaginal microbiota is closely related to HPV infection and CC [3, 4, 5, 6].

The vagina is a relatively complex microecosystem mainly composed of vaginal flora, immune mediators, periodically changing endocrine environments, and reproductive tract mucosal epithelial immune cells. The vaginal flora is of great significance in maintaining the stability of microecology, self‐purification of the vagina, and women's health [7]. Women with dysbiosis are at an increased risk of developing tumors [8].

Various tumors meet the nutritional demands of rapid proliferation through changes in metabolic patterns. Metabolic reprogramming is considered a hallmark of cancer that promotes rapid proliferation, survival, invasion, metastasis, and resistance to therapy, thereby supporting tumorigenesis [9]. When dysbiosis occurs, especially colonization by certain obligate anaerobes, enzymes and metabolites are produced, which damage the integrity of mucosal and epithelial cells and promote HPV infection and the continuous progression of cervical lesions [10]. *Gardnerella* and *Streptococcus* sp. are involved with CC at the microbial level [11].

Therefore, clarifying the role of cervicovaginal microbiota in HPV infection, cervical lesions, and CC is of great significance for prevention and treatment. The development of high‐throughput sequencing technology, 16S amplicon sequencing, and metagenomics has allowed accurate analysis of vaginal microorganisms through non‐culture methods, providing a new approach for the research, diagnosis, and treatment of HPV infections and cervical lesions [12]. However, high‐throughput sequencing is challenging due to the lack of standardization and harmonization in detection and analysis methods. These include differences in amplification regions, analysis software, calculation methods, and outcome metrics, as well as inconsistencies in the selection of clinical sample types, such as stool, urine, blood, and vaginal secretions. These limitations have led to inconsistent results and hindered the understanding of the roles of microorganisms in the development of CC [13, 14, 15]. Most studies have suggested that HPV infection and CC could lead to an increase in the diversity of the vaginal microbiome [6, 8, 16, 17, 18, 19, 20, 21, 22, 23, 24, 25]. However, other studies have shown a decrease in microbial diversity [5, 26, 27, 28, 29, 30, 31, 32, 33].

The purpose of this review is to summarize the current evidence on the application of high‐throughput sequencing in studying the cervicovaginal microbiota and perform a meta‐analysis to assess changes in microbiome diversity in patients with HPV infection and CC. Evaluation of microbial dominance, abundance, and diversity seemingly implies microbial dynamics reflected by the abundance of *Gardnerella*, *Prevotella*, *Porphyromonas*, and *Dialister* species that correlate with women's cervicovaginal conditions. This review will provide in‐depth insight into cervicovaginal microbiota associated with cervical health and disease.

## 2 Methods

### 2.1 Data Sources and Search Strategy

The systematic review was conducted following PRISMA 2020 guidelines (Checklist S1). This review was registered at PROSPERO (ID: 42023459347) (https://www.crd.york.ac.uk/prospero/). The Cochrane Library, Web of Science, PubMed, and Embase databases were searched for studies published before June 2023. There is no definite time limit for the retrieval of literature. The search strategy includes the combination of MeSH terms and free text, and the MeSH terms include “Microbiota,” “Uterine Cervical Neoplasms,” and “Human Papillomavirus Viruses” to retrieve articles related to human HPV infection, CC, and microbiome (Table S1 for search strategy).

### 2.2 Inclusion and Exclusion Criteria

The inclusion criteria were as follows: (1) definite diagnosis of HPV infection or CC, aged over 15 years; (2) types of outcomes including the results of diversity analysis or linear discriminant analysis using high‐throughput sequencing; (3) studies published in any country with human subjects; (4) case–control studies, cross‐sectional studies, retrospective and prospective cohort studies, randomized and non‐randomized controlled trials, and exploratory observational studies; and (5) research samples including cervical or vaginal secretions.

Exclusion Criteria: Pregnant women, patients with HIV infection, animal research, conference abstracts, reviews, letters to the editor, case reports, or case series.

### 2.3 Data Selection and Extraction

Two authors (W.Z. and Y.G.) independently selected the titles and abstracts of the studies and reviewed the full texts and supplementary materials. Any discrepancies were resolved by consensus or arbitration with a third author (Y.Y.). Study characteristics, microbiome diversity, and bacterial groups enriched in diseased conditions were extracted from each included study.

### 2.4 Quality Assessment

Newcastle‐Ottawa Scale was used to assess the quality of cohort and case–control studies, divided into three domains and eight items, using a star system with a full score of nine stars; the risk of bias was then considered as high (< 5 points), moderate (6–7), or low (8–9). The evaluation results of all articles are shown in Table S2.

### 2.5 Strategy of Data Synthesis

In this study, we conducted a meta‐analysis of diversity indices. Data were extracted using means and standard deviations to calculate Hedges' g and the 95% confidence intervals (CI) used in the meta‐analysis. A random‐effect model was applied to obtain pooled effect size estimates, 95% CI, and *p* values by restricting the maximum likelihood estimator. Meta‐analysis was performed using Stata MP16 software, and statistical significance was determined at *p* < 0.05. Statistical heterogeneity was assessed using *I* ^2^ statistics. Subgroup analysis was performed when the estimated values showed a heterogeneity > 70%. Publication bias was not tested because fewer than 10 studies were eligible for the meta‐analysis of each outcome.

## 3 Results

### 3.1 Study Selection

The detailed study research and selection process are shown in the PRISMA flowchart (Figure S1). A total of 2166 pieces of literature were searched. After duplicate removal and assessment of the title and abstract, a total of 64 articles were eligible for full‐text analysis, and 7 studies were selected for the quantitative synthesis.

### 3.2 Study Characteristics

The current evidence on the application of high‐throughput sequencing was examined to investigate variation in the cervicovaginal microbiota and perform a meta‐analysis to assess changes in microbiome diversity in patients with HPV infection and CC. The characteristics of the 64 studies, including 54 case–control and 10 cohort studies, were described and published between 2013 and 2023. In total, 41 studies were from Asian countries, 12 studies were from North America, 6 were from Europe, 4 studies were conducted in Africa, and 1 study was from Latin America (Table 1). Most of the studies (60 out of 64) employed 16S rRNA sequencing for the generation of microbiome data, while the remaining four studies utilized metagenomics sequencing. The 16S rRNA sequencing‐based studies considered different amplification regions such as V3–V4 (*n* = 18), V4 (*n* = 21), V1–V9 (*n* = 1), V3 (*n* = 4), V1–V2 (*n* = 2), V2–V3 (*n* = 1), V3–V5 (*n* = 2), V4–V5 (*n* = 3), and V5 (*n* = 2) and six studies omitted amplification regions. The most extensive sequencing platforms were Illumina MiSeq, Illumina HiSeq, and Illumina NovoSeq, with three studies using the Ion Torrent sequencing platform and six studies using Roche 454 sequencing.

**TABLE 1:** Characteristics of the included studies.

Study ID	Country	Study design	Study period	Total number	Intervention	Age	Disease type	Sample collection	Method to determine microbiota	Sequencing platform
Selvaraj Arokiyaraj et al. 2018	Korea	CS	2006–2013	41 N = 10 TH = 15 PH = 16	—	44.6 ± 9.0^b^ 44.4 ± 13.3 44.6 ± 13.0	HPV	Vagina	16S rRNA —	Roche 454
Audirac et al. 2016	Mexico	CCS	2008–2011	32 NCL = 17 SIL = 4 CC = 8	—	34 ± 8^b^ 40 ± 14 43 ± 11	CC	Vagina	16S rRNA V3‐V4	Roche 454
Berggrund et al. 2020	Sweden	CS	2013–2015	96 N = 32 TH = 26 PH = 38	—	36.7 ± 5.2^b^ 35.9 ± 5.2 37.5 ± 6.0	HPV	Vagina	Metagenomes	Ion Torrent
Bi et al. 2021	China	CCS	2018.01–2018.06	15 PH = 6 TH = 4 *N* = 5	—	52.17 ± 10.19^b^ 43.00 ± 4.74 40.00 ± 6.89	HPV	Vagina	16S rRNA V4	Ion S5XL
Borgogna et al. 2021	USA	CCS	2010–2011	40	—	29 ± 9.1^b^	HPV	vagina	16S rRNA V3‐V4	Roche 454
Camargo et al.2022	Colombia	CS	2007–2010	66	—	42.5 (20)^a^	HPV	Vagina	16S rRNA V4	Illumina Novaseq PE250
Chao et al. 2020	China	CCS	2018.07–2019.03	329PH =59 TH = 139 *N* = 131	—	41.68 ± 11.58^b^	HPV	Vagina	16S rRNA V4	Illumina HiSeq2500
Chao et al. 2021	China	CCS	2018.07–2019.03	272 CIN2 + =83 HPV = 86 *N* = 103	—	38.93 ± 9.59^b^	HPV HSIL	Vagina	16S rRNA V5	Illumina HiSeq2500
Chao et al.2019	China	CCS	2018.5–2018.5	151 HPV = 65 *N* = 86	—	37.86 ± 9.99^b^	HPV	Vagina	16S rRNA V5	Illumina HiSeq2500
Chen et al. 2020	China	CCS	2015.5–2016.11	229 HPV = 78 LSIL = 51 HSIL = 23 CC = 9 *N* = 68	—	43.00 ± 8.69^b^ 47.78 ± 9.63 46.00 ± 10.19 43.70 ± 10.74 56.11 ± 9.02	HPV CC	Vagina	16S rRNA V3‐V4	Illumina MiSeq
Cheng et al.2020	Sweden	CCS	—	257 HPV = 144 *N* = 113	—	14–29^c^	HPV	Vagina	16S rRNA V3‐V4	Illumina MiSeq
Cheng et al. 2020	China	CCS	2016.9–2019.6	131 LSIL = 26 HSIL = 40 CC = 32 *N* = 33	—	21–65^c^	HPV CC	Vagina	16S rRNA V4	Illumina HiSeq2000
Chorna et al. 2020	USA	CCS	—	19 HrHPV = 11 *N* = 8	—	—	HrHPV	Cervix vagina Urine	Metagenomes	—
Dareng et al. 2016	Nigeria	CCS	2012.4–2012.8	278 HrHPV = 66 *N* = 212	—	≥ 18^c^	HrHPV	Vagina	16S rRNA V4	Illumina MiSeq
Di Paola et al.2017	Italy	CS	—	73 TH = 27 PH = 28 *N* = 17	—	26–64^c^	HPV	Vagina	16S rRNA V3‐V5	Roche 454
Fang et al. 2022	China	CCS	2020.12–2021.9	40 HrHPV = 20 *N* = 20	—	33.75 ± 6.78^b^ 35.55 ± 4.12	HrHPV	Cervix	16S rRNA V3‐V4	Illumina NovaSeq
Godoy‐Vitorino et al. 2018	USA	CCS	—	62 HrHPV = 27 LrHPV = 3 Both HrHPV and LrHPV = 22 *N* = 10	—	—	HPV	Cervical vaginal rectum	16S rRNA V4	Illumina
Guo et al.2022	China	CCS	—	149 HPV + NoSIL = 40 HPV + LSIL = 28 HPV + HSIL = 51 *N* = 30	—	19–50^c^	HPV SIL	Vagina	16S rRNA V4‐V5	Illumina NovaSeq
Hu et al. 2022	China	CCS	2019	276 HPV = 94 *N* = 182	—	44.68 ± 10.94^b^	HPV	Cervix	16S rRNA V3‐V4	Illumina HiSeq
Huang et al. 2018	China	CCS	2015.4–2016.11	280 16LSIL = 30 16HSIL = 43 52LSIL = 42 52HSIL = 42 58LSIL = 42 58HSIL = 40 N = 41	—	—	HPV	Cervix	16S rRNA V4‐V5	Illumina MiSeq
Ivanov et al. 2023	Russia	CCS	—	241	Different cancer therapies CRT; RT; ST; CT	—	HPV CC	Cervix	16S rRNA V3‐V4	Illumina MiSeq
Kang et al. 2021	Korea	CCS	—	23 CC = 8 CIN = 8 *N* = 7	—	47 ± 10.2^b^ 43.4 ± 12.8 47.4 ± 5.38	CC	Vagina	16S rRNA V3	Ion Torrent
Laniewski et al. 2018	USA	CCS	—	100 N = 20 HPV = 31 LSIL = 12 HSIL = 27 CC = 10	—	39.55 ± 7.35^b^ 37.64 ± 9.38 35.08 ± 7.24 38.29 ± 8.46 38.90 ± 9.09^a^	HPV CC	Vagina	16S rRNA V4	Illumina MiSeq
Laniewski et al. 2019	USA	CCS	—	78 N = 18 HPV = 11 LSIL = 12 HSIL = 27 CC = 10	—	40.38 ± 6.98^b^ 36.36 ± 9.53 35.08 ± 7.24 38.29 ± 8.46 38.90 ± 9.09 36.36	HPV CC	Vagina	16S rRNA V4	Illumina MiSeq
Lee et al. 2013	Korea	CCS	2005–2009	68 N = 23 HPV = 45	—	—	HPV	Vagina	16S rRNA V2‐V3	Roche 454
Lee et al. 2020	Korea	CCS	—	66 CIN1− =24 CIN2 + =42	—	45.1 ± 11.7^b^	HPV	Vagina	16S rRNA V3	Ion Torrent
Lin et al. 2022	China	CCS	2018.6–2020.3	28 HPV = 23 *N* = 5	—	39.5 ± 11.1^b^ 36.6 ± 9.3	HPV	Cervix	16S rRNA V4	Illumina MiSeq
Liu et al. 2022	China	CCS	—	82 Zang = 42 Naxi = 13 Yi = 7 Bai = 4 Lisu = 6 Han = 10	—	39.7 ± 7.1^b^	HPV	Cervical vaginal rectum	16S rRNA —	Illumina MiSeq
Liu et al. 2022	China	CCS	2020.6–2021.3	115 HPV = 34 CIN = 40 CC = 41	—	—	HPV CIN CC	Cervix	Metagenomes	Illumina HiSeq2500
Liu et al. 2022	China	CCS	2016.11–2017.7	69 *N* = 16 SHPV = 31 MHPV = 22	—	35.75 ± 7.94^b^ 39.55 ± 10.05 37.82 ± 9.21	HPV	Vagina	16S rRNA V4	Illumina MiSeq
Liu et al. 2023	China	CCS	2018.7–2019.1	1015 HPV = 271 *N* = 744	—	45.1 ± 7.20^b^	HPV	Vagina	16S rRNA V4	Illumina MiSeq
Ma et al. 2023	China	CCS	2021.4–2021.7	160 NCL = 22 LSIL = 45 HSIL = 36 CC = 27 *N* = 30	—	30 (29–75)^a^	HPV SIL CC	Vagina	16S rRNA —	Illumina MiSeq
McKee et al. 2020	USA	CCS	—	308 ACC = 109 HrHPV = 110 *N* = 89	—	26 (21–39)^a^	HPV	Vagina	16S rRNA V4	Illumina MiSeq
Mei et al. 2022	China	CS	2015.1–2021.3	100 *N* = 42 PH = 28 TH = 30	—	37.29 ± 7.49^b^ 38.47 ± 7.37 38.86 ± 11.44	HrHPV	Vagina	16S rRNA V3‐V4	Illumina MiSeq
Mitra et al. 2015	UK	CCS	—	169 LSIL = 52 HSIL = 92 CC = 5 *N* = 20	—	31 ± 5.08^b^	HPV SIL CC	Vagina	16S rRNA V1‐V2	Illumina MiSeq
Mitra et al. 2020	UK	CS	2002–2007	87 PH = 45 TH = 42	—	20.5 ± 2.4^b^	HPV CIN2	Cervix	16S rRNA V1‐V2	Illumina MiSeq
Nieves‐Ramirez et al. 2021	Canada	CCS	2003.12–2006.07	228 SIL = 121 *N* = 107	—	37.26 ± 10.87^b^ 42.83 ± 7.92	HPV SIL	Vagina	16S rRNA V3	Illumina HiSeq2000
Onywera et al. 2019	South Africa	CCS	—	87 HPV = 37 *N* = 50	—	32.0 (25.0–39.0)^a^	HPV	Cervix	16S rRNA V3‐V4	Illumina MiSeq
Piyathilake et al. 2016	USA	CCS	—	430 CIN 1 = 90 CIN 2+ = 340	—	26.1 ± 4.7^b^	HPV CIN	Cervix	16S rRNA V4	Illumina MiSeq
Ritu et al. 2019	China	CS	2016.8–2017.8	133 N = 42 TH = 56 PH = 35	—	43.8 ± 8.9^b^	HPV	Cervix	16S rRNA V4	Illumina Hiseq2500
Sasivimolrattana et al. 2022	Thailand	CCS	—	48 HPV = 43 N = 5	—	37.77 ± 1.25^b^	HrHPV	Cervix	16S rRNA V1‐V9	Illumina MiSeq
Shi et al. 2022	China	CS	2015.4–2016.10	73 TH = 45 PH = 28	—	40.1 ± 11.5^b^	HPV SIL	Cervix	16S rRNA V4‐V5	Illumina MiSeq
Sims et al. 2020	Botswana	CCS	2018.7–2019.2	31 Dysplasia = 21 CC = 10	—	41.8 ± 7.5 50.7 ± 12^b^	CC	Cervix	16S rRNA V4	Illumina MiSeq
So et al. 2020	Korea	CCS	—	50 HrHPV = 40 NHrHPV = 10	—	20–50^c^	HPV	Vagina	16S rRNA V3‐V4	Illumina MiSeq
Tango et al. 2020	Korea	CCS	2006.3–2020	92 CIN2/3‐CC = 42 *N* = 50	—	45.7 ± 11.7^b^ 45.1 ± 11.6	CC	Cervix	16S rRNA —	Roche 454
Teka et al. 2023	Ethiopia	CCS	2019.10–2020.2	120 CC = 60 Dysplasia = 25 *N* = 35	—	≥ 18^c^	CC	Cervix	16S rRNA V4	Illumina MiSeq
Tosado‐Rodríguez et al. 2023	USA	CCS	2020.3–2020.10	91 *N* = 56 LSIL = 18 HSIL = 17	—	39 ± 11.3^b^	HPV SIL	Cervix	16S rRNA V4	—
Usyk et al. 2020	USA	CS	—	273 THr = 70 PHr = 170 CHr = 33	—	23.4 ± 2.8^b^ 22.6 ± 2.3 22.70 ± 2.9	HrHPV	Cervix	16S rRNA V4	Illumina MiSeq
Vikramdeo et al. 2022	USA	CCS	—	36 AA = 12 CA = 12 HIS = 12	—	35.25 ± 8.00^b^ 44.42 ± 12.32 32.92 ± 9.62	CIN CC	Cervix	16S rRNA V4	Illumina MiSeq
Wei et al. 2022	China	CCS	—	59 *N* = 10 HPV = 13 LSIL = 15 HSIL = 10 CC = 11	—	38.50 ± 5.68^b^ 37.08 ± 7.05 38.33 ± 7.35 40.50 ± 5.95 43.45 ± 5.50	HrHPV SIL CC	Vagina	16S rRNA V3‐V4	Illumina MiSeq PE300
Wei et al. 2021	China	CCS	2018.9–2018.12	60 HrHPV = 30 *N* = 30	—	36.70 ± 6.30^b^ 34.13 ± 6.43	HrHPV	Cervix Vagina	16S rRNA V3‐V4	Illumina MiSeq PE300
Wu et al. 2020	China	CCS	—	69 NILM = 31 LSIL = 22 HSIL = 16	—	38 ± 9.04^b^	HrPV SIL	Vagina	16S rRNA V3‐V4	Illumina MiSeq
Wu et al. 2021	China	CCS	2018.1–2019.12	94 CC = 13 HSIL = 31 LSIL = 10 HPV = 12 *N* = 28	—	38.6 ± 7.6^b^	HPV SIL CC	Vagina	16S rRNA V4	Illumina NovoSeq6000
Xia et al. 2022	China	CCS	2021.4–2021.10	135 *N* = 43 HrHPV = 58 LSIL = 34	—	32.27 ± 5.88^b^	HPV	Vagina	16S rRNA V3‐V4	Illumina NovoSeq6000
Xie et al. 2020	China	CCS	2018.7–2019.8	72 *N* = 27 CIN = 22 CC = 23	—	34.57 ± 2.665^b^	CIN CC	Vagina	16S rRNA V4	Illumina MiSeq
Xu et al. 2022	China	CCS	—	40 *N* = 10 LSIL = 10 HSIL CC = 10	—	48.28 ± 12.76^b^	SIL CC	Cervix Vagina	16S rRNA V3‐V4	Illumina MiSeq
Xu et al. 2023	China	CCS	—	87 HRCD = 50 *N* = 37	—	18–75^c^	HPV CIN CC	Vagina	16S rRNA V3	Illumina MiSeq
Yang et al. 2020	China	CCS	2017.2–2018.11	52 HPV16 = 27 *N* = 25	—	34.5 ± 4.5^b^ 36.9 ± 4.9	HPV	Vagina	Metagenomes	Illumina HiSeq
Zeng et al. 2023	China	CS	2016.1–2018.6	135 *N* = 45 HrHPV16/18 = 28 Non‐HrHPV16/18 = 62	Vaginal probiotics (mainly lactobacilli) interferon therapy	36 (17)^a^ 37 (18) 41.5 (10.75)	HPV	Cervix	16S rRNA —	Ion S5XL
Zeng et al. 2023	China	CCS	2019.9–2020.1	60 *N* = 15 CIN1 = 15 CIN2/3 = 15 CC = 15	—	—	HPV CIN	Vagina	16S rRNA V3‐V4	Illumina MiSeq
Zhai et al. 2021	China	CCS	2019.1–2019.12	168 *N* = 29 HrHPV = 29 LSIL = 32 HSIL = 40 CC = 38	—	40.08 ± 4.83^b^ 42.17 ± 5.18 40.63 ± 4.55 40.64 ± 5.57 42.43 ± 5.31	HrHPV SIL CC	Vagina	16S rRNA V3‐V4	Ion S5XL
Zhang et al. 2018	China	CCS	2014–2015	166 CIN1− = 64 CIN1 = 62 CIN2+ = 19 CIN3 = 21	—	—	HPV CIN	Cervix	16S rRNA V3‐V5	Illumina HiSeq2500
Zhang et al. 2022	China	CCS	2020.11–2021.5	356 *N* = 113 HPV = 159 CIN = 84	—	20–70^c^	HPV CIN CC	Cervix Vagina	16S rRNA —	Illumina Novaseq PE250
Zhang et al. 2021	China	CCS	—	100 *N* = 20 Other HrHPV = 32 HPV16/18 = 38 CC = 10	—	38.35 ± 3.72^b^ 36.75 ± 6.15 35.73 ± 6.89 38.80 ± 3.08	HPV CC	Cervix vagina	16S rRNA V3‐V4	Illumina MiSeq

Abbreviations: AA, African American women; ACC, Abnormal cervical cytology; Bai, Bai ethnic groups; CA, Caucasian American women; CC, Cervical cancer; CCS, case–control study; CHr, Hr‐HPV infection included women who developed a CIN2 or CIN3 (CIN2+) lesion; CIN, Cervical intraepithelial neoplasia; CIN1−, healthy control to CIN1; CIN2+, CIN 1‐ and CIN 2+ to cervical cancer; CRT, chemoradiotherapy; CS, cohort study; CT, combined therapy; Han, Han ethnic groups; HIS, Hispanic/Latina women; HPV, Human papillomavirus infection; HRCD, HPV‐related cervical disease, HPV infection, including CIN and cervical cancer; HrHPV, high‐risk human papillomavirus infection; HSIL, high‐grade squamous intraepithelial lesion; Lisu, Lisu ethnic groups; LSIL, low‐grade squamous intraepithelial lesion; MHPV, multiple HPV‐genotype infection; N, healthy control; Naxi, Naxi ethnic groups; NCL, non‐cervical lesions (HPV‐negative and HPV‐positive); NHrHPV, negative for high‐risk HPV; NILM, no intra‐epithelial lesion or malignancy; PH, women with persistent HPV infection; PHr, women with an infection for 2 or more years with the same type in the absence of a CIN2+ diagnosis; RT, radiotherapy; SHPV, single HPV‐genotype infection; SILs, squamous intraepithelial lesions; ST, surgical treatment; TH, women with transient HPV infection; THr, women who cleared their incident HR‐HPV infections within 1 year; Yi, Yi ethnic groups; Zang, Zang ethnic groups.

^a^ Data represent median (interquartile range).

^b^ Data represent mean ± standard deviation.

^c^ Age range.

A total of 22 studies explored the differences between microbial changes in the long‐term process of HPV infection in different degrees of cervical lesions, followed by progression to CC [5, 6, 17, 18, 19, 23, 25, 34, 35, 36, 37, 38, 39, 40, 41, 42, 43, 44, 45, 46, 47]. A total of 11 studies [3, 10, 15, 29, 48, 49, 50, 51, 52, 53, 54] have reported differences between persistent and transient HPV infections. Persistent HPV infection makes a complex microbial community, indicating that the microbial flora differs according to the infection state. High‐risk HPV (HrHPV) infection impacts the role of microbiota in the existence of cervical lesions [13, 23, 29, 32, 46, 53, 55, 56, 57, 58]. Four studies investigated whether infection with a single HPV genotype or multiple HPV genotypes was associated with diverse communities composed of vaginal microbiota [25, 54, 59, 60]. Different treatment methods were applied for CC and compared differences in cervicovaginal microorganisms after treatment [17]. Interferons combined with probiotics (*Lactobacillus*) were used to treat the HPV infection and attempted to determine the mechanism by which *Lactobacillus* protects the vagina [54].

### 3.3 Microbiome Diversity

Alpha diversity included Shannon (*n* = 53), Simpson (*n* = 24), CHAO (*n* = 23), observed species (*n* = 12), ACE (*n* = 8), and phylogenetic diversity whole tree (*n* = 6) indices. Beta diversity included principal component analysis (*n* = 5), principal coordinate analysis (*n* = 40), and other methods (*n* = 2) (Table 2).

**TABLE 2:** Description of microbial alpha and beta diversity.

Study ID	Software	Alpha							Beta			
		Alpha richness and diversity	Shannon	Simpson	Chao	Observed_species	ACE	PD whole tree	Beta diversity	PCA	PCoA	Others
Selvaraj Arokiyaraj et al. 2018	Mothur	Higher PH and TH than N	⊕	─	⊕	─	─	─	Difference between N, TH, and PH	─	⊕	─
Audirac et al. 2016	QIIME	Higher CC and SIL groups than NCL	⊙	─	─	─	─	⊕	No difference between CC, SIL groups and NCL	─	⊕	─
Berggrund et al.2020	QIIME	1. No significant difference between N, TH and PH at baseline	⊙	─	─	─	─	─	NR	─	─	─
		2. No significant difference between TH and PH at second sample	⊙									
Bi et al. 2021	QIIME	Higher PH than TH and N	⊕	⊕	⊕	⊕	⊕	─	NR	─	─	─
Camargo et al. 2022	QIIME	1. Higher low VL than high VL and medium VL	⊕	⊕	─	─	⊕	─	1. Difference between low VL, high VL and medium VL	─	⊕	─
		2. Higher increase VL than equal VL	⊙	⊙	─	─	⊕	─	2. No difference between increase VL, equal VL and decrease VL	─	⊙	─
Chao et al. 2020	—	No difference between PH, TH, and N	⊙	─	─	─	─	─	1. Difference between PH and TH	─	⊕	─
									2. Difference between PH and N	─	⊕	─
									3. No difference between TH and N	─	⊙	─
Chao et al. 2021	—	1. Higher CIN2+ than HPV	⊕									
		2. Higher CIN2+ than N	⊕	─	─	─	─	─	Difference between CIN2+, HPV and N	─	⊕	─
		3. No difference between P and N	⊙									
Chen et al. 2020	Mothur R	1. Higher HPV‐positive (HPV; LSIL; HSIL; CC) than N	⊕	─	⊕	─	─	─	Difference between HPV, LSIL, HSIL, CC and N	─	⊕	─
		2. CC were highest than HPV, LSIL, HSIL	⊕									
Cheng et al.	R	Higher HPV than N	⊕	─	⊕	─	─	─	No difference between HPV and N	─	⊙	─
Cheng et al. 2020	—	1. Higher HPV than N	⊕	─	─	─	─	⊙	NR	─	─	─
		2. Higher CC than LSIL, HSIL, N	⊕	─	─	─	─	⊕				
Chorna et al. 2020	QIIME	1. Higher HrHPV than N at vaginal	─	─	⊕	─	─	─	No difference between HrHPV and N at vaginal and cervical	─	⊙	─
		2. No difference between HrHPV and N at cervical	─	─	⊙	─	─	─				
Dareng et al. 2016	QIIME	─	─	─	─	─	─	─	Difference between HrHPV and N	─	⊕	─
Di Paola et al.2017	R	Higher PH, TH than N	⊙	─	⊙	⊙	─	─	NR	─	─	─
Fang et al. 2022	QIIME	Higher HrHPV than N	⊕	⊕	─	─	─	─	Difference between HrHPV and N	─	⊕	─
Godoy‐Vitorino et al. 2018	R	Higher CIN3 than CIN1 at cervix and vaginal	⊕	─	─	─	─	─	Difference between cervix, vaginal and anal	─	⊕	─
Guo et al.2022	R	1. Higher HSIL than LSIL	⊕	─	─	─	─	─	Differences between N, HPV + NoSIL, LSIL andHPV+HSIL	─	⊕	─
		2. No differences between HPV + LSIL and HPV + NoSIL	⊙	─	─	─	─	─				
		3. No difference between HPV + NoSIL and N	⊙									
Hu et al. 2022	QIIME	Higher HPV than N	─	─	⊕	─	─	─	NR	─	─	─
Huang et al. 2018	QIIMER	Higher SILs than N	⊕	─	⊕	─	⊕	─	Difference between SILs and N	─	⊕	─
Ivanov et al. 2023	QIIME	1. Higher CC than N	⊕	─	─	⊕	─	─				
		2. Higher HSIL than N	⊕						NR	─	─	─
		3. Lower in PT+ than PT—	⊙	─	─	⊙	─	─				
Kang et al. 2021	QIIME	Higher CC, CIN than N	⊙	⊙	─	─	─	─	Difference between CC, CIN and N	─	⊕	─
Laniewski et al. 2018	USEARCH	NR	─	─	─	─	─	─	No difference between HPV, CIN1, CIN2/3, CC and N	─	⊙	─
Laniewski et al. 2019	USEARCH	NR	─	─	─	─	─	─	Difference between HPV, LSIL, HSIL, CC and N	⊕	─	─
Lee et al. 2013	QIIME	NR	─	─	─	─	─	─	NR	─	─	─
Lee et al. 2020	QIIME VSEARCH	NR	─	─	─	─	─	─	No difference between CIN1− and CIN2+	⊙	─	─
Lin et al. 2022	R	Higher HPV than N	⊕	⊕	⊙	─	⊙	─	NR	─	─	─
Liu et al. 2022	Mothur	1. Similar among all samples from the six ethnic groups	─	─	─	─	─	─	1. Difference between rectal and cervical, vaginal	⊕	─	─
		2. Highest in the rectal and was higher in cervical than in vaginal	─						2. No difference between cervical and vaginal	⊙	─	─
									3. No difference between HPV and N at rectal, cervical, vaginal	⊙	─	─
Liu et al. 2022	R	1. Higher CIN and CC than HPV	⊕	─	⊕	─	─	─	Difference between CIN and CC, HPV	─	⊙	─
		2. No differences between CIN and CC	─	─	⊙	─	─	─				
Liu et al. 2022	Mothur	1. No differences between SHPV, MHPV and N at genus level	⊙	⊙	⊙	─	⊙	─	Difference between MHPV and SHPV, N	─	⊕	─
		2. Higher SHPV than MHPV and N	⊙	⊙	⊕	─	⊙	─				
Liu et al. 2023	QIIME	1. Higher BV & HPV than N	⊕	─	─	⊕	─	─				
		2. N, non‐BV and HPV, BV and HPV− to BV and HPV+, an increasing trend of alpha diversities	─	─	─	─	─	─	1. Difference between BV and HPV and N	─	⊕	─
		3. Higher SHPV than N	⊕	─	─	⊕	─	─	2. Difference between MHPV and SHPV	─	⊕	─
		4. Higher MHPV than N	⊕	─	─	⊕	─	─				
		5. Higher MHPV than SHPV	⊙	─	─	⊕	─	─				
Ma et al. 2023	QIIME	Higher CC than LSIL, NV+ and N	⊕	⊕	─	─	─	─	NR	─	─	─
McKee et al. 2020	Mothur	No difference between ACC, HrHPV and N	⊙	⊙	─	─	─	─	Difference between ACC, HrHPV and N	─	─	⊕
Mei et al. 2022	Mothur R	1. Higher N than TH	─	─	⊕	─	─	─				
		2. No difference between PH and N	─	─	⊙	─	─	─	Difference between PH, TH and N	─	⊕	─
		3. No difference between PH and TH	─	─	⊙	─	─	─				
Mitra et al. 2015	MothurR	Higher CC, HSIL, LSIL than N	⊙	⊙	─	⊙	─	─	NR	─	─	─
Mitra et al. 2020	MothurR	Higher PH than TH	─	⊙	─	─	─	─	NR	─	─	─
Nieves‐Ramirez et al. 2021	Mothur	1. Higher SIL than N	⊕	─	⊕	─	─	─	1. Difference between SIL and N	─	⊕	─
		2. No difference between HPV_Lesion HPV‐no_Lesion	⊙	─	⊙	─	─	─	2. No difference between HPV_Lesion HPV‐no_Leson	─	⊕	─
		3. Higher HPV_Lesion than N	⊕	─	⊕	─	─	─	3. Difference between HPV and N	─	⊕	─
		4. Higher HPV than N	⊕	─	⊕	─	─	─				
Onywera et al. 2019	QIIME	1. No difference between HPV and N	⊙						1. No difference between HPV and N	─	⊙	─
		2. No difference between HrHPV and N	⊙	⊙	─	─	─	─	2. No difference between HrHPV and N	─	⊙	─
Piyathilake et al. 2016	QIIME	No difference between CIN1 and CIN2+	─	─	─	─	─	─	No difference between CIN1 and CIN2+	─	⊙	─
Ritu et al. 2019	QIIME	1. Higher N than HPV	⊙	⊙	⊕	⊕	─	⊙				
		2. No difference between TH and PH	⊙	⊙	⊙	⊙	─	⊙	NR	─	─	─
Sasivimolrattana et al. 2022	—	Higher NLD than LD	⊕	─	─	─	─	─	Difference between NLD and LD	─	─	─
Shi et al. 2022	R	No difference between PH and TH	⊙	─	⊙	─	─	─	No difference between PH and TH	─	⊙	─
Sims et al. 2020	USEARCH	1. Higher CC than Dysplasia	⊕						1. Difference between CC and Dysplasia	─	⊕	─
		2. Higher CIN3 than CIN2	⊕	─	─	─	─	─	2. Difference between CIN3 and CIN2	─	⊕	─
So et al. 2020	QIIME	Higher HPV than N	⊕	─	─	─	─	─	Difference between CC, CIN and N	─	⊕	─
Tango et al. 2020	USEARCH	Higher CIN2/3‐CC than N	⊙	⊙	⊕	─	─	─	No difference between CIN2/3‐CC and N	─	⊙	─
Teka et al. 2023	QIIME	1. No difference between HPV and N	⊙	⊙	─	─	─	─	1. Difference between HPV and N	─	⊕	─
		2. Higher CC than Dysplasia and N	⊕	⊕	─	─	─	─	2. Difference between CC and Dysplasia, N	─	⊕	─
Tosado‐Rodríguez et al. 2023	QIIME	1. No difference between HPV and N	⊙	─	⊙	─	─	─	Difference between HSIL and LSIL	─	⊕	─
		2. Higher HSIL than N	⊕	─	⊕	─	─	─				
Usyk et al. 2020	VSEARCH	1. No difference between THr and PHr and CHr at visit 1	⊙	─	─	─	─	─	NR	─	─	─
		2. Higher CHr than PHr and THr at visit 2	⊕	─	─	─	─	─				
Vikramdeo et al. 2022	USEARCH	1. Higher CIN than N	⊙	─	─	⊕	─	─				
		2. No difference between HPV and N	⊙	─	─	⊙	─	─	NR	─	─	─
Wei et al. 2022	QIIMER	1. Higher CC than N	⊕									
		2. Higher HSIL than N	⊕	⊕	─	─	─	─	1. Difference between CC and N	─	⊕	─
		3. Higher LSIL than N	⊕									
		4. Higher HPV than N	⊕									
		5. No difference between CC and HSIL, LSIL, HPV	⊙	⊙	─	─	─	─	2. No difference between HSIL and LSIL	─	⊙	─
Wei et al. 2021	QIIME	No difference between HPV and N	⊙	─	⊙	⊙	─	─	Difference between HPV and N	⊕	─	─
Wu et al. 2020	—	Higher NILM than LSIL and HSIL	⊙	⊙	─	─	─	─	NR	─	─	─
Wu et al. 2021	QIIME	1. Higher CC than N	⊕									
		2. Higher HSIL than N	⊕	⊕	─	─	─	─	Difference between CC, HSIL, LSIL, HPV and N	─	⊕	─
		3. Higher LSIL than N	⊕									
		4. Higher N than HPV	⊕									
Xia et al. 2022	QIIMER	1. Higher HrHPV than N	⊕	─	⊕	─	─	─	No difference between HrHPV and LSIL, N	─	⊙	─
		2. Higher HrHPV than LSIL	⊙	─	⊕	─	─	─				
Xie et al. 2020	QIIME	1. No difference between CIN and CC	⊙	⊙	─	─	─	─	Difference between CIN, CC and N	─	⊕	─
		2. Higher CIN and CC than N	⊙	⊙	─	─	─	─				
Xu et al. 2022	QIIME	Higher CC than N	⊕	⊕	─	─	─	─	Difference between CC, HSIL, LSIL and N	─	⊕	─
Xu et al. 2023	QIIME	Higher HRCD than N	⊕	⊕	─	─	⊙	─	No difference between HRCD and N	─	⊙	⊙
Yang et al. 2020	R	No difference between HPV16 and N	⊙	⊙	─	─	─	─	No difference between HPV16 and N	⊙	─	─
Zeng et al. 2023	QIIME VSEARCH	1. Higher HrHPV16/18 than N	⊙	⊙	⊕	⊕	⊕	─	1. No difference between HrHPV16/18, HrHPV16/18 and N	─	⊙	─
		2. Higher MHPV and twoHPV than N	⊙	⊙	⊙	⊙	⊙	⊙	2. Higher MHPV and twoHPV than N	─	⊙	─
		3. Higher PH than TH	⊙	⊙	⊕	⊕	⊙	⊙				
Zeng et al. 2023	USEARCHMothur	1. Higher CC than N	⊕	⊕	⊕	⊕	─	⊕	1. No difference between CIN1 and N	─	⊙	─
		2. Higher CC than CIN1	⊙	⊙	⊕	⊕	─	⊕	2. Difference between CC and CIN1, CIN2/3 N	─	⊕	─
		3. Higher CC than CIN2/3	⊕	⊕	⊕	⊕	─	⊕	3. No difference between CIN2/3 and CC	─	⊙	─
Zhai et al. 2021	QIIME	1. Higher N than HrHPV	⊕	⊕	⊕	⊕	⊕	⊙	Difference between CC, HSIL, LSIL, HrHPV and N	─	⊕	─
		2. No difference between SIL and CC	⊙	⊙	⊙	⊙	⊙	⊙				
Zhang et al. 2018	UPARSEQIIME	1. No difference between CIN1− and CIN2+	⊙	─	─	─	─	─	1. No difference between CIN1− and CIN2+	─	⊙	─
		2. No difference between CIN1− and CIN3	⊙	─	─	─	─	─	2. No difference between CIN1− and CIN3			
Zhang et al. 2022	MothurQIIME	1. Higher CIN than N	⊕	─	⊕	─	─	─	No difference between HPV, CIN and N	─	⊕	─
		2. Higher HPV than N	⊕	─	⊕	─	─	─				
		3. Higher SHPV than N	⊕	─	⊕	─	─	─				
		4. Higher MHPV than N	⊕	─	⊕	─	─	─				
		5. Higher CIN than N	⊕	─	⊕	─	─	─				
		6. Higher HrHPV than N	⊕	─	⊕	─	─	─				
		7. Higher LrHPV than N	⊕	─	⊕	─	─	─				
Zhang. et al. 2021	CutadaptPEAR Prinseq Usearch Uchime Muscle FastTree Mothur R	1. Higher cervial N than vaginal N	⊕									
		2. Higher cervial Other Hrhpv than vaginal Other Hrhpv	⊕	─	─	─	─	─	Difference between the cervical and vaginal microbiota	─	⊕	─
		3. Higher cervial Hrhpv than vaginal Hrhpv	⊕									
		4. No difference between cervial CC and vaginal CC	⊙	─	─	─	─	─				

*Note:* NR, not report; ⊕ represented *p* < 0.05, ⊙ represented *p* > 0.05.

Abbreviations: BV, bacterial vaginosis; CHr, HrHPV infection included women who developed a CIN2 or CIN3 (CIN2+) lesion; LD, lactobacilli‐dominated; LT, large tumor; MHPV, multiple HPV‐genotype infection; NLD, non‐lactobacilli‐dominated; P, HPV infection without cervical neoplasia; PHr, women with an infection for 2 or more years with the same type in the absence of a CIN2+ diagnosis; PT+, surgery; SHPV, single HPV‐genotype infection; ST, small tumor; THr, women who cleared their incident HrHPV infections within 1 year; twoHPV, infected by two HPV‐genotype infection; VL, viral load.

### 3.4 Changes of Alpha Diversity in Patients Infected With HPV

Nine studies [8, 16, 20, 23, 25, 35, 36, 61, 62] showed that microbial diversity was higher in patients infected with HPV than in healthy controls, two studies [6, 51] showed the opposite result, and five studies [5, 21, 30, 32, 63] found no difference. No correlation was observed between alpha diversity and HPV infection in South African women [30], Ethiopian women [21], Hispanic/Latina (HIS), African Americans (AA) [5], and Hispanics living in Puerto Rico [63]. In contrast, among Asian women, although statistical uniformity is not complete, there is a consistent trend that the invasion of HPV increases the diversity of cervical and vaginal microorganisms [16, 20, 23, 25, 32, 35, 36, 61, 62]. Therefore, region and race cannot be ignored in such studies. Similarly, patients infected with HrHPV have a higher microbial diversity than the normal Asian population [25, 54, 56, 64], and only one study found no significant difference in microbial diversity between the two groups [46]. In addition, the sample collection site was one of the factors in microbial diversity; vaginal samples from patients with HrHPV had higher microbial diversity than those from healthy controls [64]; however, there was no difference in the cervix [13].

Another interesting phenomenon is observed in patients with persistent and transient HPV infections (HPV clearance status). Cervicovaginal microbial diversity shows no significant difference between those with persistent and transient HPV infection [29, 48, 51, 52, 53], but five studies showed the diversity of patients with persistent HPV infection was more complex than that of patients with transient HPV infection [10, 15, 49, 50, 54], although not statistically uniform. These results were affected by age and duration of HPV infection; the population in these cohort studies ranged from 18 to 70 years, and the duration of the studies ranged from 6 months to 2 years. Therefore, age‐dependent variations in microbiome makeup need to be considered while evaluating the correlation between HPV infection type/severity and microbiome diversity and comparing the same across various age groups.

### 3.5 Changes of Alpha Diversity in Patients With CC

The microbial diversity of patients with CC was not only higher than that of HPV‐infected patients [18, 35] but also significantly higher than that of healthy controls [6, 17, 21, 22, 23, 24, 34, 35, 36, 37]. In a study with participants of two age groups, when the age was > 50 years, the diversity of patients with CC was higher than that of healthy people. When the age was less than 50 years, the significant difference between the two groups was absent [31]. The diversity of CC samples from different sites (including cervix and vagina) showed no differences [47]. Although the microbial diversity of patients with cervical dysplasia and cervical intraepithelial neoplasia (squamous intraepithelial lesions) was also increased, the observed differences were not statistically significant [18, 23, 43, 46]. Moreover, the microbial diversity of patients with CC was higher than that of patients with cervical dysplasia [19, 21] and cervical intraepithelial neoplasia [24, 35, 36].

### 3.6 Meta‐Analyses

**FIGURE 1:**
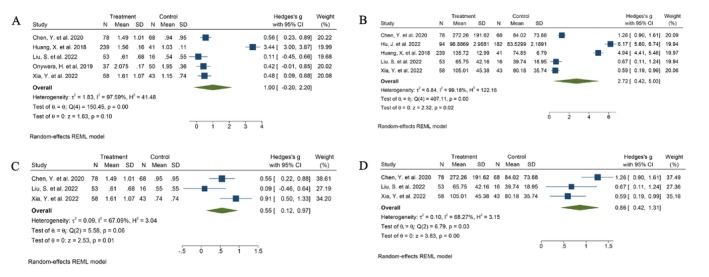
Forest plot of the differences in alpha diversity between patients with HPV infection and healthy control: (A) Shannon index, (B) CHAO index; Subgroup analysis at vagina (C) Shannon index, (D) CHAO index.

Shannon and Chao indices were used for quantitative analysis (*n* = 7). The Shannon index evaluates species evenness, and the Chao index focuses more on species richness. The Shannon index showed that the microbial diversity between patients infected with HPV and healthy controls was statistically insignificant (Hedges' *g* = 1.00, 95% CI 0.20 to 2.20, *I* ^2^ = 97.59%). The Chao index indicated statistical significance (Hedges' *g* = 2.72, 95% CI 0.42 to 5.03, *I* ^2^ = 99.18%) (Figure 1A,B). It can be inferred that the total number of HPV‐infected species was higher than that in healthy people. However, the difference in species evenness was not obvious.

**FIGURE 2:**
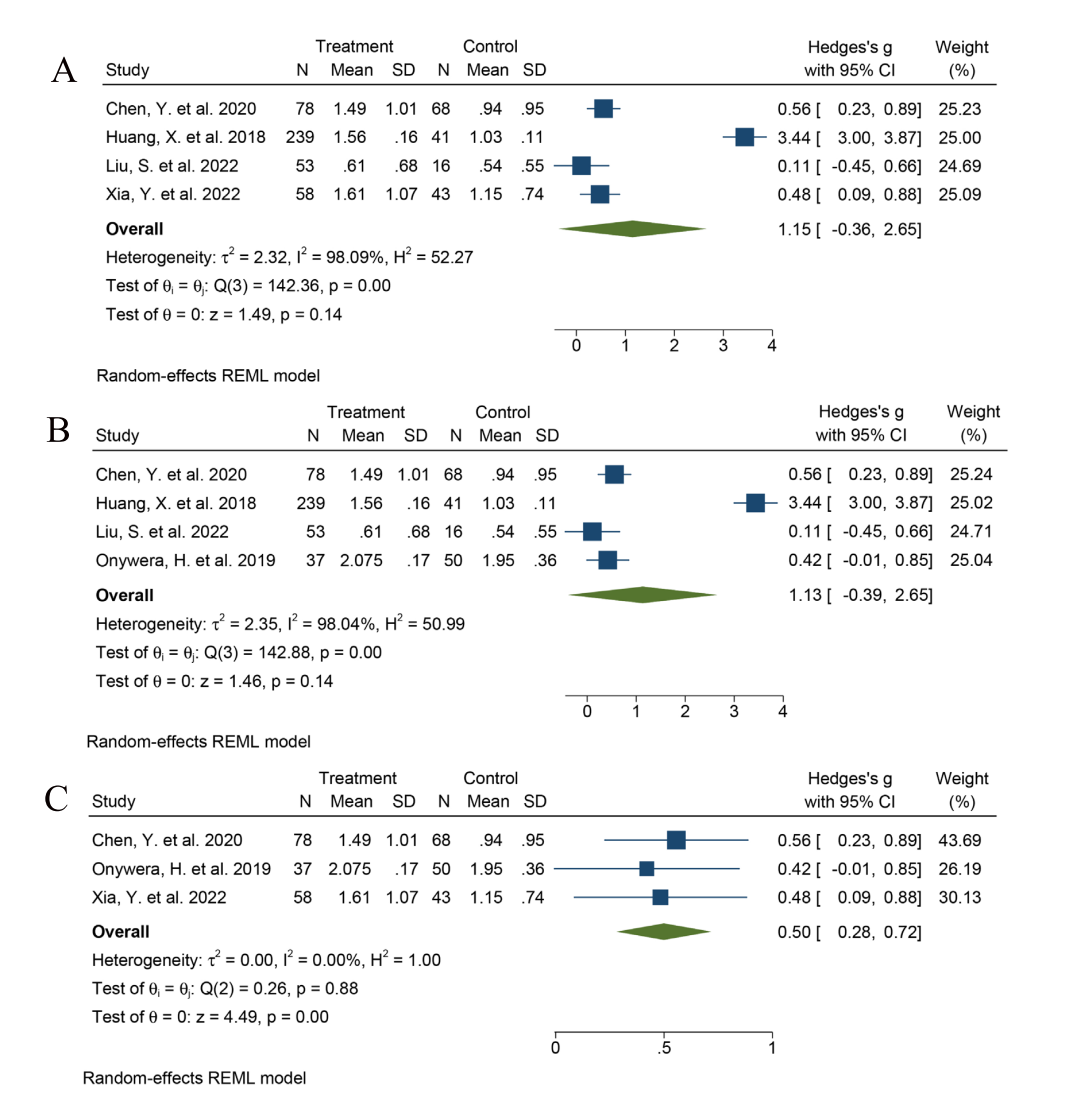
Subgroup analysis of Shannon index between patients with HPV infection and healthy control: (A) China, (B) Illumina MiSeq, (C) primer pairs.

The subgroup analysis was conducted according to the sample collection regions, and the I^2^ value decreased (Shannon index: Hedges' *g* = 0.55, 95% CI 0.12 to 0.97, *I* ^2^ = 67.09%, CHAO index: Hedges' *g* = 0.86, 95% CI 0.42 to 1.31, *I* ^2^ = 68.27%) with significant heterogeneity. The results showed that the Shannon and Chao index were statistically significant in the vagina (Figure 1C,D). Subgroup analysis was performed according to the country, and no statistical difference between patients infected with HPV and the healthy controls was observed (Shannon index: Hedges' *g* = 1.15, 95% CI −0.36 to 2.65, *I* ^2^ = 98.09%) (Figure 2A). Subgroup analysis was performed based on the sequencing platform (Figure 2B) and primer pair (variable region) selection (Figure 2C). When the primer pair was selected on V3–V4 the statistical difference (Shannon index: Hedges' *g* = 0.50, 95% CI 0.28 to 0.72, *I* ^2^ = 0.00%) was observed. This suggests that the site of sample collection and the choice of the primer pair may be responsible for heterogeneity.

**FIGURE 3:**
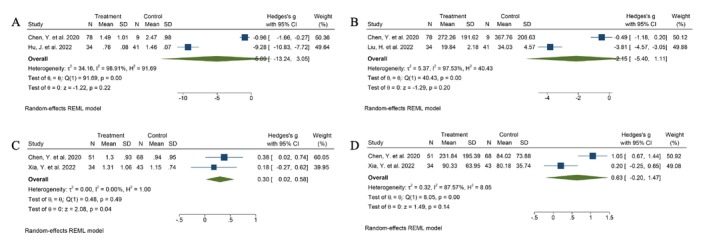
Forest plot of the differences in alpha diversity between patients with HPV infection and cervical cancer: (A) Shannon index, (B) CHAO index; alpha diversity between patients with low‐grade squamous intraepithelial lesion and healthy control: (C) Shannon index, (D) CHAO index.

The microbial diversity between patients with HPV infection and CC was statistically insignificant (Shannon index: Hedges' *g* = −5.09, 95% CI −13.24 to 3.05, *I* ^2^ = 98.91%, Chao index: Hedges' *g* = −2.15, 95% CI −5.40 to 1.11, *I* ^2^ = 97.53%) (Figure 3A,B). Chao index (Hedges' *g* = 0.63, 95% CI 0.20 to 1.47, *I* ^2^ = 87.57%) and Shannon index (Hedges' *g* = 0.30, 95% CI 0.02 to 0.58, *I* ^2^ = 0.00%) revealed significant differences in microbial diversity between patients with low‐grade cervical lesions and healthy controls (Figure 3C,D). These results suggest that cervical lesions can disrupt the structure and function of the residing microbiome.

### 3.7 Beta Diversity

Eight studies reported differences between the microorganisms in patients with HPV and HrHPV infections and healthy controls [8, 21, 28, 32, 48, 55, 56, 60], and a considerable number of reports also showed no difference between the two groups [13, 16, 25, 30, 33, 45, 54, 64, 65]. Two studies reported differences in beta diversity between patients with persistent and transient HPV infections [15, 66], while two showed no difference [48, 52]. The cervicovaginal microbiome exhibits differences between single HPV and multiple HPV infections [29, 54, 59, 60].

The beta diversity of patients with CC was significantly different from that of healthy controls [6, 20, 21, 22, 23, 28, 31, 34, 37, 43, 44, 46, 67], similar to the differences observed in alpha diversity; only three studies reported no difference [42, 45, 66]. The diversity of microorganisms undergoes dynamic changes during the development of CC [4, 6, 8, 18, 19, 20, 21, 24, 26, 27, 35, 37, 39, 43, 44, 46]. However, some studies have pointed out that there is not necessarily a strong causal relationship between cervicovaginal microbial diversity and the severity of cervical lesions [11, 23, 24, 25, 38, 45, 68, 69]. Overall, the non‐overlapping results indicate the need for studies with more homogeneous and larger cohort sizes in order to infer a statistically significant correlation of microbial features (like diversity) with disease phenotypes.

### 3.8 Bacterial Groups Enriched in Diseased Condition

**FIGURE 4:**
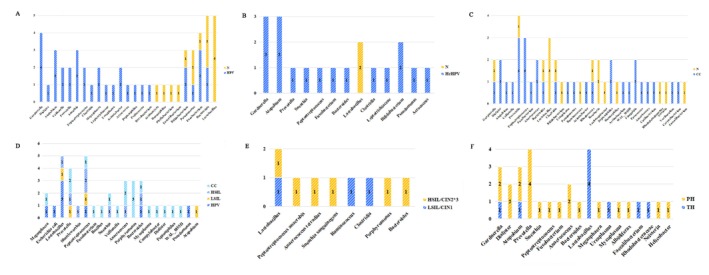
Number of times that different bacteria have enriched for different diseases at the genus level. CC, cervical cancer; CIN1, low‐grade cervical intraepithelial neoplasia; CIN2*3, high‐grade cervical intraepithelial neoplasia; HPV, human papillomavirus infection; HrHPV, high‐risk human papillomavirus infection; HSIL, high‐grade squamous intraepithelial lesion; LSIL, low‐grade squamous intraepithelial lesion; N, healthy control; PH, persistent HPV infections; TH, transient HPV infections.

Table S3 shows the enrichment of different bacteria in diseased conditions, and Figure 4 shows the number of studies that reported an enrichment of a bacteria in diseased conditions at the genus level.

Patients with HPV and HrHPV infections are enriched with similar bacterial species (Figure 4A,B), including *Gardnerella* [30, 33, 56, 61, 64, 70], *Sneathia* [30, 49, 62, 71], *Atopobium* [30, 56, 61, 62, 70], *Prevotella* [25, 55, 62], *Veillonella* [18, 70], *Peptostreptococcus* [33, 62, 69], *Megasphaera* [35, 63], *Acinetobacter* [72], *Bifidobacterium* [61], *Fusobacterium* [51, 62, 71], *Ureaplasma* [51], *Dialister* [70], and *Pseudomonas* [30, 49]. *Gardnerella*, as reported by the highest number of studies to be enriched in HPV infection, can be investigated further regarding its role in the etiology of HPV infection and its potential as a biomarker for improved screening and diagnosis.

For patients with CC, the biomarkers in different studies did not completely overlap (Figure 4C), and most pathogenic and enriched bacteria included *Porphyromonas* [6, 18, 35], *Fusobacterium* and *Prevotella* [6, 17, 18, 40, 42, 66], *Mycoplasma* [6], *Megasphaera* [6], *Campylobacter*, *Dialister* [6, 32, 40, 66], *Peptoniphilus*, *Peptostreptococcus* [40], *Anaerococcus* [6], *WAL_1855D* [6], *Bacillus*, *Sneathia* [17, 35, 40], *Bacteroides* [18, 40], *Veillonella* [40, 42], *Saccharimonas*, *Streptococcus*, *Ureaplasma* [42], *Gardnerella* [32], and *Atopobium* [32]. Compared with the healthy controls, the proportions of *Prevotella*, *Fusobacterium*, and *Dialister* increased with the progression of cervical lesions [18, 73], whereas *Gardnerella* was less prominent in CC. *Porphyromonas* [6, 18, 31, 35, 71] can be used as a marker for screening CC compared with different degrees of cervical lesions (Figure 4D,E). *Lactobacillus* was dominant during transient HPV infection (Figure 4F) and had an unstable microbiome environment [9, 46, 48, 69]. *Prevotella* [10, 49, 50, 51], *Dialister* [48, 51], *Atopobium* [15, 49], *Gardnerella*, and *Anaerococcus* [48, 50] were the most common bacteria in patients with persistent HPV infection. These bacteria may play a role in disrupting the epithelial barrier [10], thus favoring persistent HPV infection or transmission. Different types of *Lactobacilli* in the cervicovaginal microbiota show significant differences in their correlation with HPV [18, 50, 51, 54].

Both persistent and transient infections are associated with subgroups of *Lactobacillus*, and the function of different types of *Lactobacilli* is usually considered a protective mechanism. However, subgroups of *Lactobacillus* have been reported to be closely associated with increased vaginal inflammation and an increased probability of transformation into bacterial vaginosis. Therefore, different types of *Lactobacilli*, such as *Lactobacillus iners* , *L. crispatus* , and *L. gasseri* , have bidirectional roles in cervicovaginal health and disease [18, 74].

### 3.9 Risk of Bias Assessment

The quality assessment results of these studies are presented in Table S2. Among 10 cohort studies, three scored 9 points, one scored 8 points, and the others were of moderate quality: four scored 7 points, and two scored 6 points. In some studies, the follow‐up time was short, or the number of follow‐ups was missing. Among the identified 54 case–control studies, 15 studies were of high quality: three scored 9 points, 12 studies scored 8 points, and 30 studies were of moderate quality: 12 scored 7 points, and 18 scored 6 points; the other studies scored 5 and 4 points. “Comparability of Cases and Controls on the Basis of the Design or Analysis” and “Non‐Response rate” were not identified in most studies.

## 4 Discussion

This systematic review and meta‐analysis evaluated studies on microbiota changes in patients infected with HPV and with CC using high‐throughput sequencing. The normal cervicovaginal microbiota structure is simple, less diverse, and dominated by *Lactobacillus*. Microbial dysbiosis and increased diversity are more likely to allow HPV invasion, which is a risk factor for CC and is related to the severity of cervical lesions. The differences in country/population, age, microbial sequencing technology, primer pair, and sample collection site (vaginal and cervical secretions) significantly impacted the composition of microorganisms.

The composition of the cervicovaginal microbiota is dynamic; it varies depending on the female reproductive cycle, hormonal changes, oral contraceptives, sexual activity, lactation, and smoking [7] and exhibits highly variable associations with race, geography, and individual genetic factors [13, 65]. Studies on cervicovaginal microecology have focused only on the disease and neglected the impact of external factors. However, environmental and physiological changes can greatly affect patients infected with HPV. While this study describes changes in the vaginal microbiota following HPV infection in different ethnic groups, there is limited research comparing the microbiota of patients from different backgrounds within the same study to allow for direct comparison and control of confounding factors. Future studies should compare racial and ethnic groups to better characterize the influence of race/ethnicity on the cervicovaginal microbiota.

HPV infection causes extreme changes in the composition and abundance of bacteria within a short interval [53], constantly shifting and evolving bacterial communities over time and possibly eventually returning to their original state [75]. However, the duration of the process remains unclear. A positive association was found between vaginal pH and HPV positivity in women (18–34 years), but the differences were not statistically significant in women aged > 65 years [76]. Changes in vaginal pH are associated with the vaginal microenvironment; therefore, age is also a factor that cannot be ignored.

Subgroup analysis revealed that the sampling location was an important contributing factor. Although the anatomical definition, the low female reproductive tract (vagina and cervix) is closely linked [77], most studies prefer to refer to samples as “cervicovaginal” rather than discuss “cervical” and “vaginal” samples. However, different parts of the female genital tract have versatile microbiota [47]. The sampling location and sampling method are important in determining the individual vaginal microbial community composition [78]. Subsequent studies should be described separately as much as possible, or standardized sampling locations and methods should ensure the homogeneity of the research results. Further studies are needed to determine which site plays a greater role in the disease.

Differences in existing sequencing methods are also an influencing factor, as the selection of primer pairs (variable regions) is more important than the sequencing platform. When the primer pair V3–V4 (341F–785R) is selected, the bacteria coverage is the highest. A study has also shown that the primer pair V3–V4 slightly outperformed the other combinations, regardless of the reference database, and is a justified choice for human gut samples [12]. With the development of metagenomic and V1–V9 full‐length sequencing, microbiome information can be mined in more detail at very high sequencing depths. However, owing to the high economic cost of such sequencing methods, the number of studies is limited, and their advantages are not clear.

Only seven studies were included in the quantitative analysis; the statistical power of our meta‐analysis was also reduced by the large amount of missing data for published studies. Six studies were from China, which had great heterogeneity and could not represent global data.

The biomarkers at different stages of cervical lesions also differed, which may be related to the different reference controls. The relative abundance of bacterial genera identified in the same group under different primer pairs was different, leading to significant changes in biomarkers identified by the LefSe statistical tool [79]. *Gardnerella* was more likely to appear during HPV infection [37, 56, 61, 70], and *Prevotella*, *Dialister*, and *Porphyromonas* were closely related to advanced cancer. Biogenic Amines [80] and metabolites of *Prevotella*, *Dialister*, and *Peptostreptococcus* can protect bacteria from acidic conditions while also reducing the growth rate of *Lactobacillus* and promoting the establishment and maintenance of the Community State Type IV (CST IV, Low‐Lactobacillus) microbiota, which is not found in *Gardnerella*. In addition, these bacteria mediate carcinogenesis, including generating pro‐inflammatory conditions, immune suppression, and suppression of apoptosis [6].

### 4.1 Limitations and Future Suggestions

The meta‐analysis performed in the current review was limited by data heterogeneity, which included patients with HPV infection and CC from different countries and diverse data generation and analysis approaches. The number of studies included in this meta‐analysis was limited. In the future, with an increase in the study population and the deepening of the research direction, more high‐quality studies with large sample sizes should be included to draw more reliable and convincing conclusions. All of the included primary studies were observational studies.

Despite the limitations, the HPV vaccine is the best way to prevent CC. One study has shown that the diversity and dominant organisms of the vaginal microbiome do not change after women receive three doses of the HPV‐16/18 AS04‐adjuvanted vaccine (Cervarix) [81]. However, due to the small sample size of this study, it cannot be used as sufficient evidence to prove the impact of vaccines on microorganisms. Current approaches to regulating the microbiome for potentially positive health outcomes include therapeutic HPV vaccines based on probiotic engineering [82]. Mucosal vaccines can directly target the mucosa in the reproductive tract to deliver antigens and induce a potent local protective immune response [83]. It has been shown that mucosal vaccines based on *Lactobacillus lactis* transfected with HPV E6/E7 hold promise in preventing HPV infection and halting the progression of CC [82, 84]. Decreases in vaginal microbiota by antibiotics, replenishment of the microbiota by vaginal microbiota transplantation (VMT), and supplementation of the microbiota with prebiotics or probiotics may impact response to cancer treatment [77].

## 5 Conclusions

The microbial diversity in patients with HPV infection is highly unstable, and the impact of confounding factors on their study results must be considered. The microecological environment of the cervix and vagina changes continuously after cervical lesions occur. As long as cervical epithelial lesions develop, the microbial diversity increases, and the complexity of the microecological environment intensifies with the progression of cervical lesions. Distinct bacterial markers were identified at different stages of the cervical lesions. *Gardnerella* was prevalent in the stage of HPV infection; however, its proportion decreased significantly after the occurrence of CC. The proportions of *Prevotella*, *Bacillus*, and *Porphyromonas* were also found to be increased. The lack of high‐quality randomized controlled trial data makes it difficult to provide strong evidence of a causal relationship between microbial HPV infection and cervical lesions, and its mechanism has not been deeply explored. Therefore, additional randomized controlled trials are needed to confirm the relationship between the cervical vaginal microenvironment and the progression of cervical lesions.

## Author Contributions

Conceptualization, methodology, formal analysis, investigation, data curation, writing‐original draft preparation, and project administration: W.Z. Methodology, investigation, writing‐review and editing: Y.G. Formal analysis, writing‐original draft preparation: L.Y. Investigation, writing‐review and editing: Q.Y. Methodology, investigation, writing‐review and editing: J.W. Conceptualization, investigation, writing‐review and editing, and supervision: Y.Y. Conceptualization, methodology, investigation and supervision: B.L. All authors have read and agreed to the published version of the manuscript.

## Ethics Statement

The authors have nothing to report.

## Consent

The authors have nothing to report.

## Conflicts of Interest

The authors declare no conflicts of interest.

## Supporting information


**Data S1.** Checklist S1: PRISMA 2020 Checklist.

**Figure S1.** Description of the selection of the included studies following a PRISMA flow diagram.

**Table S1.** Search strategies.

**Table S2.** Quality Assessment.

**Table S3.** Bacterial groups enriched in diseased condition.

## Data Availability

Data sharing is not applicable to this article as no datasets were generated or analysed during the current study.
