# Supplementary material for: Changes of Microbiome in Human Papillomavirus Infection and Cervical Cancer: A Systematic Review and Meta‐Analysis
Source: Cancer Rep (Hoboken). 2025 Jun 2;8(6):e70246. doi: 10.1002/cnr2.70246 (PMC12127774; doi:10.1002/cnr2.70246)
Supplement: Supplementary file 2 — Figure S1. Description of the selection of the included studies following a PRISMA flow diagram. [file CNR2-8-e70246-s005.doc]

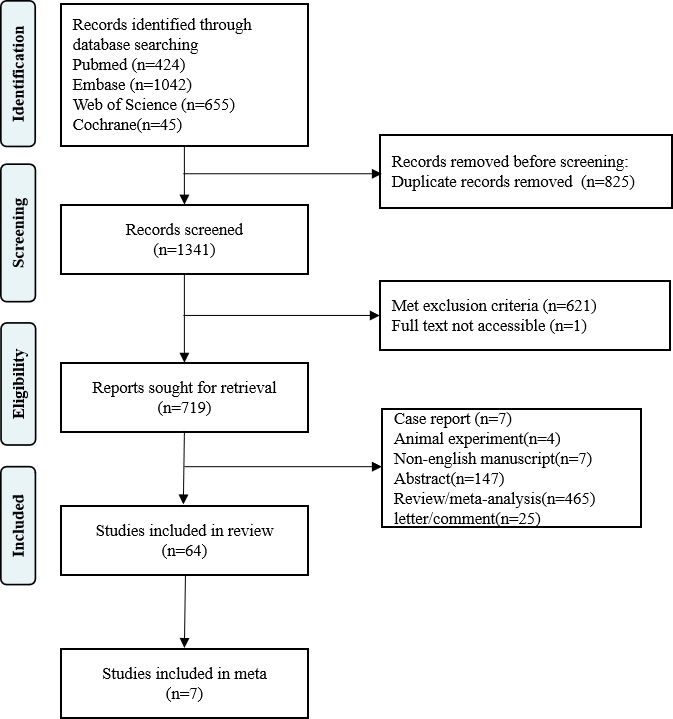


**Figure 1.** Description of the selection of the included studies following a PRISMA flflow diagram.
