# Supplementary material for: Changes of Microbiome in Human Papillomavirus Infection and Cervical Cancer: A Systematic Review and Meta‐Analysis
Source: Cancer Rep (Hoboken). 2025 Jun 2;8(6):e70246. doi: 10.1002/cnr2.70246 (PMC12127774; doi:10.1002/cnr2.70246)
Supplement: Supplementary file 3 — Table S1. Search strategies. [file CNR2-8-e70246-s003.docx]

Supplement Table S1 Search strategies.

***Pubmed***

| Search | Query | Result |
| --- | --- | --- |
| 8 | (((((((((((((((((("Microbiota"[Mesh]) OR (microbiota[Title/Abstract])) OR (Microbiotas[Title/Abstract])) OR (Microbial Community[Title/Abstract])) OR (Community, Microbial[Title/Abstract])) OR (Microbial Communities[Title/Abstract])) OR (Microbial Community Composition[Title/Abstract])) OR (Community Composition, Microbial[Title/Abstract])) OR (Composition, Microbial Community[Title/Abstract])) OR (Microbial Community Compositions[Title/Abstract])) OR (Microbial Community Structure[Title/Abstract])) OR (Community Structure, Microbial[Title/Abstract])) OR (Microbial Community Structures[Title/Abstract])) OR (Microbiome[Title/Abstract])) OR (Microbiomes[Title/Abstract])) OR (Human Microbiome[Title/Abstract])) OR (Human Microbiomes[Title/Abstract])) OR (Microbiome, Human[Title/Abstract])) AND ((((((((((((((((((((((((("Uterine Cervical Neoplasms"[Mesh]) OR (Uterine Cervical Neoplasms[Title/Abstract])) OR (Cervical Neoplasm, Uterine[Title/Abstract])) OR (Neoplasm, Uterine Cervical[Title/Abstract])) OR (Uterine Cervical Neoplasm[Title/Abstract])) OR (Neoplasms, Cervical[Title/Abstract])) OR (Cervical Neoplasms[Title/Abstract])) OR (Cervical Neoplasm[Title/Abstract])) OR (Neoplasms, Cervix[Title/Abstract])) OR (Cervix Neoplasm[Title/Abstract])) OR (Neoplasm, Cervix[Title/Abstract])) OR (Cervix Neoplasms[Title/Abstract])) OR (Cancer of the Uterine Cervix[Title/Abstract])) OR (Cancer of the Cervix[Title/Abstract])) OR (Cervical Cancer[Title/Abstract])) OR (Cancer, Cervical[Title/Abstract])) OR (Cervical Cancers[Title/Abstract])) OR (Uterine Cervical Cancer[Title/Abstract])) OR (Cancer, Uterine Cervical[Title/Abstract])) OR (Cervical Cancer, Uterine[Title/Abstract])) OR (Uterine Cervical Cancers[Title/Abstract])) OR (Cancer of Cervix[Title/Abstract])) OR (Cervix Cancer[Title/Abstract])) OR (Cancer, Cervix[Title/Abstract])) OR (((((((((((((((("Human Papillomavirus Viruses"[Mesh]) OR (Human Papillomavirus Viruses[Title/Abstract])) OR (Human Papillomavirus Virus[Title/Abstract])) OR (Papillomavirus Virus, Human[Title/Abstract])) OR (Virus, Human Papillomavirus[Title/Abstract])) OR (Human Papillomavirus[Title/Abstract])) OR (Human Papillomaviruses[Title/Abstract])) OR (HPV, Human Papillomavirus Viruses[Title/Abstract])) OR (Human Papilloma Virus[Title/Abstract])) OR (Human Papilloma Viruses[Title/Abstract])) OR (Papilloma Virus, Human[Title/Abstract])) OR (Virus, Human Papilloma[Title/Abstract])) OR (HPV Human Papillomavirus[Title/Abstract])) OR (HPV Human Papillomaviruses[Title/Abstract])) OR (Human Papillomavirus, HPV[Title/Abstract])) OR (Human Papillomaviruses, HPV[Title/Abstract]))) | 424 |
| 7 | ((((((((((((((((("Microbiota"[Mesh]) OR (microbiota[Title/Abstract])) OR (Microbiotas[Title/Abstract])) OR (Microbial Community[Title/Abstract])) OR (Community, Microbial[Title/Abstract])) OR (Microbial Communities[Title/Abstract])) OR (Microbial Community Composition[Title/Abstract])) OR (Community Composition, Microbial[Title/Abstract])) OR (Composition, Microbial Community[Title/Abstract])) OR (Microbial Community Compositions[Title/Abstract])) OR (Microbial Community Structure[Title/Abstract])) OR (Community Structure, Microbial[Title/Abstract])) OR (Microbial Community Structures[Title/Abstract])) OR (Microbiome[Title/Abstract])) OR (Microbiomes[Title/Abstract])) OR (Human Microbiome[Title/Abstract])) OR (Human Microbiomes[Title/Abstract])) OR (Microbiome, Human[Title/Abstract]) | 163,122 |
| 6 | "Microbiota"[Mesh] | 74,005 |
| 5 | (((((((((((((((((((((((("Uterine Cervical Neoplasms"[Mesh]) OR (Uterine Cervical Neoplasms[Title/Abstract])) OR (Cervical Neoplasm, Uterine[Title/Abstract])) OR (Neoplasm, Uterine Cervical[Title/Abstract])) OR (Uterine Cervical Neoplasm[Title/Abstract])) OR (Neoplasms, Cervical[Title/Abstract])) OR (Cervical Neoplasms[Title/Abstract])) OR (Cervical Neoplasm[Title/Abstract])) OR (Neoplasms, Cervix[Title/Abstract])) OR (Cervix Neoplasm[Title/Abstract])) OR (Neoplasm, Cervix[Title/Abstract])) OR (Cervix Neoplasms[Title/Abstract])) OR (Cancer of the Uterine Cervix[Title/Abstract])) OR (Cancer of the Cervix[Title/Abstract])) OR (Cervical Cancer[Title/Abstract])) OR (Cancer, Cervical[Title/Abstract])) OR (Cervical Cancers[Title/Abstract])) OR (Uterine Cervical Cancer[Title/Abstract])) OR (Cancer, Uterine Cervical[Title/Abstract])) OR (Cervical Cancer, Uterine[Title/Abstract])) OR (Uterine Cervical Cancers[Title/Abstract])) OR (Cancer of Cervix[Title/Abstract])) OR (Cervix Cancer[Title/Abstract])) OR (Cancer, Cervix[Title/Abstract])) OR (((((((((((((((("Human Papillomavirus Viruses"[Mesh]) OR (Human Papillomavirus Viruses[Title/Abstract])) OR (Human Papillomavirus Virus[Title/Abstract])) OR (Papillomavirus Virus, Human[Title/Abstract])) OR (Virus, Human Papillomavirus[Title/Abstract])) OR (Human Papillomavirus[Title/Abstract])) OR (Human Papillomaviruses[Title/Abstract])) OR (HPV, Human Papillomavirus Viruses[Title/Abstract])) OR (Human Papilloma Virus[Title/Abstract])) OR (Human Papilloma Viruses[Title/Abstract])) OR (Papilloma Virus, Human[Title/Abstract])) OR (Virus, Human Papilloma[Title/Abstract])) OR (HPV Human Papillomavirus[Title/Abstract])) OR (HPV Human Papillomaviruses[Title/Abstract])) OR (Human Papillomavirus, HPV[Title/Abstract])) OR (Human Papillomaviruses, HPV[Title/Abstract])) | 142,124 |
| 4 | ((((((((((((((("Human Papillomavirus Viruses"[Mesh]) OR (Human Papillomavirus Viruses[Title/Abstract])) OR (Human Papillomavirus Virus[Title/Abstract])) OR (Papillomavirus Virus, Human[Title/Abstract])) OR (Virus, Human Papillomavirus[Title/Abstract])) OR (Human Papillomavirus[Title/Abstract])) OR (Human Papillomaviruses[Title/Abstract])) OR (HPV, Human Papillomavirus Viruses[Title/Abstract])) OR (Human Papilloma Virus[Title/Abstract])) OR (Human Papilloma Viruses[Title/Abstract])) OR (Papilloma Virus, Human[Title/Abstract])) OR (Virus, Human Papilloma[Title/Abstract])) OR (HPV Human Papillomavirus[Title/Abstract])) OR (HPV Human Papillomaviruses[Title/Abstract])) OR (Human Papillomavirus, HPV[Title/Abstract])) OR (Human Papillomaviruses, HPV[Title/Abstract]) | 52,329 |
| 3 | "Human Papillomavirus Viruses"[Mesh] | 7,025 |
| 2 | ((((((((((((((((((((((("Uterine Cervical Neoplasms"[Mesh]) OR (Uterine Cervical Neoplasms[Title/Abstract])) OR (Cervical Neoplasm, Uterine[Title/Abstract])) OR (Neoplasm, Uterine Cervical[Title/Abstract])) OR (Uterine Cervical Neoplasm[Title/Abstract])) OR (Neoplasms, Cervical[Title/Abstract])) OR (Cervical Neoplasms[Title/Abstract])) OR (Cervical Neoplasm[Title/Abstract])) OR (Neoplasms, Cervix[Title/Abstract])) OR (Cervix Neoplasm[Title/Abstract])) OR (Neoplasm, Cervix[Title/Abstract])) OR (Cervix Neoplasms[Title/Abstract])) OR (Cancer of the Uterine Cervix[Title/Abstract])) OR (Cancer of the Cervix[Title/Abstract])) OR (Cervical Cancer[Title/Abstract])) OR (Cancer, Cervical[Title/Abstract])) OR (Cervical Cancers[Title/Abstract])) OR (Uterine Cervical Cancer[Title/Abstract])) OR (Cancer, Uterine Cervical[Title/Abstract])) OR (Cervical Cancer, Uterine[Title/Abstract])) OR (Uterine Cervical Cancers[Title/Abstract])) OR (Cancer of Cervix[Title/Abstract])) OR (Cervix Cancer[Title/Abstract])) OR (Cancer, Cervix[Title/Abstract]) | 112,634 |
| 1 | "Uterine Cervical Neoplasms"[Mesh] | 84,984 |

***Embase***

| Search | Query | Result |
| --- | --- | --- |
| #11 | #9 AND #10 | 1042 |
| #10 | #3 OR #6 | 199,903 |
| #9 | #7 OR #8 | 254,736 |
| #8 | microflora:ab,ti OR microbiota:ab,ti OR microbiotas:ab,ti OR 'microbial community':ab,ti OR 'community, microbial':ab,ti OR 'microbial communities':ab,ti OR 'microbial community composition':ab,ti OR 'community composition, microbial':ab,ti OR 'composition, microbial community':ab,ti OR 'microbial community compositions':ab,ti OR 'microbial community structure':ab,ti OR 'community structure, microbial':ab,ti OR 'microbial community structures':ab,ti OR microbiome:ab,ti OR microbiomes:ab,ti OR 'human microbiome':ab,ti OR 'human microbiomes':ab,ti OR 'microbiome, human':ab,ti | 187,534 |
| #7 | 'microflora'/exp | 192,039 |
| #6 | #4 OR #5 | 72,650 |
| #5 | 'wart virus':ab,ti OR 'human papillomavirus viruses':ab,ti OR 'human papillomavirus virus':ab,ti OR 'papillomavirus virus, human':ab,ti OR 'virus, human papillomavirus':ab,ti OR 'human papillomavirus':ab,ti OR 'human papillomaviruses':ab,ti OR 'hpv, human papillomavirus viruses':ab,ti OR 'human papilloma virus':ab,ti OR 'human papilloma viruses':ab,ti OR 'papilloma virus, human':ab,ti OR 'virus, human papilloma':ab,ti OR 'hpv human papillomavirus':ab,ti OR 'hpv human papillomaviruses':ab,ti OR 'human papillomavirus, hpv':ab,ti OR 'human papillomaviruses, hpv':ab,ti | 60329 |
| #4 | 'wart virus'/exp | 43664 |
| #3 | #1 OR #2 | 158,067 |
| #2 | 'uterine cervix tumor':ab,ti OR 'uterine cervical neoplasms':ab,ti OR 'cervical neoplasm, uterine':ab,ti OR 'neoplasm, uterine cervical':ab,ti OR 'uterine cervical neoplasm':ab,ti OR 'neoplasms, cervical':ab,ti OR 'cervical neoplasms':ab,ti OR 'cervical neoplasm':ab,ti OR 'neoplasms, cervix':ab,ti OR 'cervix neoplasm':ab,ti OR 'neoplasm, cervix':ab,ti OR 'cervix neoplasms':ab,ti OR 'cancer of the uterine cervix':ab,ti OR 'cancer of the cervix':ab,ti OR 'cervical cancer':ab,ti OR 'cancer, cervical':ab,ti OR 'cervical cancers':ab,ti OR 'uterine cervical cancer':ab,ti OR 'cancer, uterine cervical':ab,ti OR 'cervical cancer, uterine':ab,ti OR 'uterine cervical cancers':ab,ti OR 'cancer of cervix':ab,ti OR 'cervix cancer':ab,ti OR 'cancer, cervix':ab,ti | 88058 |
| #1 | 'uterine cervix tumor'/exp | 146,343 |

*****Web of science*****

| Search | Query | Result |
| --- | --- | --- |
| #1 | TS=(Microbiota) | 154566 |
| #2 | AB=(Microbiota OR Microbiotas OR Microbial Community OR Community, Microbial OR Microbial Communities OR Microbial Community Composition OR Community Composition, Microbial OR Composition, Microbial Community OR Microbial Community Compositions OR Microbial Community Structure OR Community Structure, Microbial OR Microbial Community Structures OR Human Microbiome OR Human Microbiomes OR Microbiome, Human OR Microbiome OR Microbiomes) | 195309 |
| #3 | #2 OR #1 | 236547 |
| #4 | TS=(Uterine Cervical Neoplasms) | 93561 |
| #5 | AB=(Uterine Cervical Neoplasms OR Cervical Neoplasm, Uterine OR Neoplasm, Uterine Cervical OR Uterine Cervical Neoplasm OR Neoplasms, Cervical OR Cervical Neoplasms OR Cervical Neoplasm OR Neoplasms, Cervix OR Cervix Neoplasm OR Neoplasm, Cervix OR Cervix Neoplasms OR Cancer of the Uterine Cervix OR Cancer of the Cervix OR Cervical Cancer OR Cancer, Cervical OR Cervical Cancers OR Uterine Cervical Cancer OR Cancer, Uterine Cervical OR Cervical Cancer, Uterine OR Uterine Cervical Cancers OR Cancer of Cervix OR Cervix Cancer OR Cancer, Cervix) | 90348 |
| #6 | #4 OR #5 | 138343 |
| #7 | TS=(Human Papillomavirus Viruses) | 59308 |
| #8 | AB=(Human Papillomavirus Viruses OR Human Papillomavirus Virus OR Papillomavirus Virus, Human OR Virus, Human Papillomavirus OR Human Papillomavirus OR Human Papillomaviruses OR HPV, Human Papillomavirus Viruses OR Human Papilloma Virus OR Human Papilloma Viruses OR Papilloma Virus, Human OR Virus, Human Papilloma OR HPV Human Papillomavirus OR HPV Human Papillomaviruses OR Human Papillomavirus, HPV OR Human Papillomaviruses, HPV) | 49777 |
| #9 | #8 OR #7 | 72222 |
| #10 | #9 OR #6 | 182999 |
| #11 | #10 AND #3 | 655 |

*****Cochrone*****

| Search | Query | Result |
| --- | --- | --- |
| #1 | MeSH descriptor: [Uterine Cervical Neoplasms] explode all trees | 2951 |
| #2 | (Uterine Cervical Neoplasms or Cervical Neoplasm, Uterine or Neoplasm, Uterine Cervical or Uterine Cervical Neoplasm or Neoplasms, Cervical or Cervical Neoplasms or Cervical Neoplasm or Neoplasms, Cervix or Cervix Neoplasm or Neoplasm, Cervix or Cervix Neoplasms or Cancer of the Uterine Cervix or Cancer of the Cervix or Cervical Cancer or Cancer, Cervical or Cervical Cancers or Uterine Cervical Cancer or Cancer, Uterine Cervical or Cervical Cancer, Uterine or Uterine Cervical Cancers or Cancer of Cervix or Cervix Cancer or Cancer, Cervix):ti,ab,kw (Word variations have been searched) | 7670 |
| #3 | #1 or #2 | 7670 |
| #4 | MeSH descriptor: [Human Papillomavirus Viruses] explode all trees | 267 |
| #5 | (Human Papillomavirus Viruses or Human Papillomavirus Virus or Papillomavirus Virus, Human or Virus, Human Papillomavirus or Human Papillomavirus or Human Papillomaviruses or HPV, Human Papillomavirus Viruses or Human Papilloma Virus or Human Papilloma Viruses or Papilloma Virus, Human or Virus, Human Papilloma or HPV Human Papillomavirus or HPV Human Papillomaviruses or Human Papillomavirus, HPV or Human Papillomaviruses, HPV):ti,ab,kw (Word variations have been searched) | 3061 |
| #6 | #4 or #5 | 3061 |
| #7 | #3 or #6 | 9263 |
| #8 | MeSH descriptor: [Microbiota] explode all trees | 1751 |
| #9 | (Microbiota or Microbiotas or Microbial Community or Community, Microbial or Microbial Communities or Microbial Community Composition or Community Composition, Microbial or Composition, Microbial Community or Microbial Community Compositions or Microbial Community Structure or Community Structure, Microbial or Microbial Community Structures or Microbiome or Microbiomes or Human Microbiome or Human Microbiomes or Microbiome, Human):ti,ab,kw (Word variations have been searched) | 10076 |
| #10 | #8 or #9 | 10081 |
| #11 | #7 and #10 | 45 |
