# Supplementary material for: Changes of Microbiome in Human Papillomavirus Infection and Cervical Cancer: A Systematic Review and Meta‐Analysis
Source: Cancer Rep (Hoboken). 2025 Jun 2;8(6):e70246. doi: 10.1002/cnr2.70246 (PMC12127774; doi:10.1002/cnr2.70246)
Supplement: Supplementary file 4 — Table S2. Quality Assessment. [file CNR2-8-e70246-s001.docx]

Supplement Table S2 Quality Assessment.

*Cohort Studies*

| Study ID | Representativeness of the exposed cohort | Selection of non exposed cohort | Ascertainment of exposed | Demonstration that outcomes of interest was not present at start of study | Comparability of cohorts on the basis of the design or analysis | Assessment of outcome | Was follow-up long enough for outcomes to occur | Adequacy of follow up of cohorts | Scores |
| --- | --- | --- | --- | --- | --- | --- | --- | --- | --- |
| Selvaraj Arokiyaraj et al.2018 | ★ | ★ | ★ |  | ★ | ★ | ★ |  | 6 |
| Berggrund, M.et al.2020 | ★ | ★ | ★ | ★ | ★ | ★ |  | ★ | 7 |
| Camargo, M. et al.2022 | ★ | ★ | ★ | ★ | ★ | ★ | ★ | ★ | 8 |
| Di Paola, M. et al.2017 | ★ | ★ | ★ | ★ | ★ | ★ | ★ |  | 7 |
| Mei, L. et al. 2022 | ★ | ★ | ★ | ★ | ★★ | ★ | ★ | ★ | 9 |
| Mitra, A. et al. 2020 | ★ | ★ | ★ | ★ | ★★ | ★ | ★ | ★ | 9 |
| Ritu, W. et al. 2019 | ★ | ★ | ★ | ★ | ★ |  | ★ | ★ | 7 |
| Shi, W. et al. 2022 | ★ | ★ | ★ | ★ | ★ | ★ |  |  | 6 |
| Usyk, M. et al. 2020 | ★ | ★ | ★ | ★ | ★★ | ★ | ★ | ★ | 9 |
| Zeng, M. et al. 2023 | ★ | ★ | ★ | ★ | ★ | ★ | ★ |  | 7 |

*Case-control Studies*

| Study ID | Is the case definition adequate? | Representativeness of the Cases | Selection of Controls | Definition of Controls | Comparability of Cases and Controls on the Basis of the Design or Analysis | Ascertainment of exposure | Same method of ascertainment for cases and controls | Non-Response rate | Total scores |
| --- | --- | --- | --- | --- | --- | --- | --- | --- | --- |
| Audirac et al.2016 | ★ | ★ | ★ | ★ | ★ |  | ★ |  | 6 |
| Bi, Q.et al.2021 | ★ |  | ★ | ★ | ★ |  | ★ | ★ | 6 |
| Borgogna et al.2021 | ★ | ★ | ★ | ★ | ★ | ★ | ★ | ★ | 8 |
| Chao, X. et al. 2020 | ★ | ★ | ★ | ★ | ★ |  |  |  | 5 |
| Chao, X. et al. 2021 | ★ | ★ | ★ | ★ | ★ |  | ★ |  | 6 |
| Chao, X.-P. et al.2019 | ★ | ★ | ★ | ★ | ★ | ★ | ★ |  | 7 |
| Chen, Y. et al. 2020 | ★ | ★ | ★ | ★ | ★ |  | ★ |  | 6 |
| Cheng, L. et al.2020 | ★ | ★ | ★ | ★ | ★ | ★ | ★ | ★ | 8 |
| Cheng, W. et al. 2020 | ★ | ★ | ★ | ★ | ★ |  | ★ | ★ | 7 |
| Chorna, N. et al. 2020 | ★ | ★ | ★ | ★ |  | ★ | ★ |  | 6 |
| Dareng, E. O. et al. 2016 | ★ | ★ | ★ | ★ |  | ★ |  | ★ | 6 |
| Fang, B. et al. 2022 | ★ | ★ | ★ | ★ | ★ | ★ | ★ | ★ | 8 |
| Godoy-Vitorino, F. et al. 2018 | ★ | ★ | ★ | ★ | ★ | ★ |  | ★ | 7 |
| Guo, C. et al. 2022 | ★ | ★ | ★ | ★ | ★ |  | ★ | ★ | 8 |
| Hu, J. et al. 2022 | ★ | ★ | ★ | ★ | ★ | ★ | ★ |  | 7 |
| Huang, X. et al. 2018 | ★ | ★ | ★ | ★ | ★ | ★ | ★ | ★ | 8 |
| Ivanov, M. K. et al. 2023 | ★ | ★ | ★ | ★ |  | ★ | ★ | ★ | 7 |
| Kang, G. U. et al. 2021 | ★ | ★ | ★ | ★ | ★ | ★ | ★ |  | 7 |
| Laniewski, P. et al. 2018 | ★ | ★ | ★ |  | ★ | ★ |  |  | 5 |
| Laniewski, P. et al. 2019 | ★ | ★ | ★ |  | ★ | ★ |  |  | 5 |
| Lee, J. E. et al. 2013 | ★ | ★ | ★ | ★ | ★ |  | ★ |  | 6 |
| Lee, Y. H. et al. 2020 | ★ | ★ | ★ | ★ | ★ | ★ |  | ★ | 7 |
| Lin, W. et al. 2022 | ★ | ★ | ★ | ★ | ★ | ★ |  |  | 6 |
| Liu, C. J. et al. 2022 | ★ | ★ | ★ | ★ | ★ | ★ | ★ | ★ | 8 |
| Liu, H. et al. 2022 | ★ | ★ | ★ | ★ | ★★ | ★ | ★ | ★ | 9 |
| Liu, S. et al. 2022 | ★ | ★ | ★ | ★ | ★ | ★ |  | ★ | 7 |
| Liu, Y. et al. 2023 | ★ | ★ | ★ | ★ | ★★ | ★ | ★ | ★ | 9 |
| Ma, Y. et al. 2023 | ★ | ★ | ★ | ★ | ★ | ★ | ★ | ★ | 8 |
| McKee, K. S. et al. 2020 | ★ | ★ | ★ | ★ | ★ |  | ★ |  | 6 |
| Mitra, A. et al. 2015 | ★ | ★ | ★ | ★ | ★ | ★ | ★ |  | 7 |
| Nieves-Ramirez, M. E. et al. 2021 | ★ | ★ | ★ | ★ | ★ | ★ | ★ | ★ | 8 |
| Onywera, H. et al. 2019 | ★ | ★ | ★ | ★ | ★ |  | ★ |  | 6 |
| Piyathilake, C. J. et al. 2016 | ★ | ★ |  | ★ | ★ | ★ |  |  | 5 |
| Sasivimolrattana, T. et al. 2022 | ★ |  | ★ | ★ | ★ |  |  |  | 4 |
| Sims, T. T. et al. 2020 | ★ | ★ | ★ | ★ | ★ | ★ |  | ★ | 7 |
| So, K. A. et al. 2020 | ★ | ★ |  | ★ | ★ |  |  |  | 4 |
| Tango, C. N. et al. 2020 | ★ | ★ |  | ★ | ★ | ★ | ★ |  | 6 |
| Teka, B. et al. 2023 | ★ | ★ | ★ | ★ | ★ | ★ | ★ | ★ | 8 |
| Tosado-Rodríguez, E. et al. 2023 | ★ | ★ | ★ | ★ | ★ |  |  |  | 5 |
| Vikramdeo, K. S. et al. 2022 | ★ | ★ | ★ | ★ | ★ | ★ | ★ | ★ | 8 |
| Wei, B. et al. 2022 | ★ | ★ | ★ | ★ | ★ |  | ★ |  | 6 |
| Wei, Z. T. et al. 2021 | ★ | ★ | ★ | ★ | ★ | ★ |  |  | 6 |
| Wu, M. et al. 2020 | ★ | ★ | ★ | ★ | ★ |  | ★ |  | 6 |
| Wu, S. et al. 2021 | ★ | ★ | ★ | ★ | ★ | ★ | ★ | ★ | 8 |
| Xia, Y. et al. 2022 | ★ | ★ | ★ | ★ | ★ | ★ |  |  | 6 |
| Xie, Y. et al. 2020 | ★ | ★ |  | ★ | ★ | ★ |  |  | 5 |
| Xu, H. et al. 2022 | ★ | ★ | ★ | ★ | ★ | ★ |  |  | 6 |
| Xu, X. et al. 2023 | ★ | ★ | ★ | ★ | ★ | ★ | ★ | ★ | 8 |
| Yang, Q. et al. 2020 | ★ | ★ | ★ | ★ | ★ | ★ | ★ | ★ | 9 |
| Zeng, W. et al. 2023 | ★ | ★ |  | ★ |  | ★ |  |  | 4 |
| Zhai, Q. et al. 2021 | ★ | ★ | ★ | ★ | ★ |  | ★ |  | 6 |
| Zhang, C. et al. 2018 | ★ | ★ | ★ | ★ | ★ | ★ | ★ |  | 7 |
| Zhang, Y. et al. 2022 | ★ | ★ | ★ | ★ |  | ★ | ★ |  | 6 |
| Zhang, Z. et al. 2021 | ★ | ★ | ★ | ★ | ★ | ★ | ★ |  | 7 |
