# Supplementary material for: Changes of Microbiome in Human Papillomavirus Infection and Cervical Cancer: A Systematic Review and Meta‐Analysis
Source: Cancer Rep (Hoboken). 2025 Jun 2;8(6):e70246. doi: 10.1002/cnr2.70246 (PMC12127774; doi:10.1002/cnr2.70246)
Supplement: Supplementary file 5 — Table S3. Bacterial groups enriched in diseased condition. [file CNR2-8-e70246-s002.docx]

Supplement Table S3 Bacterial groups enriched in diseased condition.

| Study ID | LDA Score Threshold | Case(abundance) | | | | | | | | | Control(abundance) | |  |
| --- | --- | --- | --- | --- | --- | --- | --- | --- | --- | --- | --- | --- | --- |
| Selvaraj Arokiyaraj et al.2018 | >2.0 | **PH:**Mycoplasmataceae | | | **TH:**Eubacterium_eligens, Ureapiasma_urealyticum, Gardnerella_vaginalis, Microcystic_aeruginosa, Gemella_asaccharolytica | | | | | | Lactobacillus_crispatus, Corynebacterium_sundsvallense, Fackdamia_hominis, Fusobacterium_naviforme, Actinobaculum_schaali, Helcococcus_ovis | |  |
| Bi, Q.et al.2021 | >4.0 | **PH:**Proteobacteria, Alphaproteobacteria, Actinobacteria,unidentified_Actinobacteria, Clostridia, Rhizobiales, Bacteroidetes, Gammaproteobacteria, Rhizobiaceae, Phyllobacterium, Veillonellaceae, Bacteroidales, unidentified_ Clostridiales, Prevotellaceae, Sphingomonadaceae, Anaerococcus, Sphingomonas_leidyi, Prevotella, Porphyromonadaceae, Bacteroides_coprocola, Burkholderiaceae, Clostridium_ papyrosolvens, Dialister, Peptoniphilus | | **TH:**Lactobacillus_ iners | | | | | | | Lactobacillus, Lactobacillaceae , Lactobacillales, Bacilli , Firmicutes | |  |
| Borgogna et al.2021 | >2.0 | **HPV:**Gardnerella vaginalis, Eggerthella , Dialister sp. type 2, Gemella, Atopobium Spp.*, Veillonellaceae, Aerococcus christensenil, Peptoniphilus asaccharolyticus | | | | | | | | | Bifidobacteriaceae, Atopobium vaginae, Lactobacillus jensenii, Lactobacillus vaginalis, Lactobacillus crispatus | |  |
| Chao, X. et al. 2020 | >4.0 | **PH:**Prevotella | | **TH:**Lactobacillus iners | | | | | | | Bacteroidetes , Bacteroidia , Bacteroidetes , Prevotellaceae | |  |
| Chao, X. et al. 2021 | >4.0 | **CIN2*3:**Xanthomonadaceae, Stenotrophomnonas | | | | | | | | | Bacteroidia, Bacteroidetes, Bacteroidales | |  |
| Chao, X.-P. et al.2019 | ─ | **HPV:**Acinetobacter, Bacteroides_plebeius, Acinetobacter_lwoffii, Lactobacillus_oleohominis | | | | | | | | | Bacteroides, Faecalibacterium, Bacteroides_uniformis, Bacteroides_ovatus, Bacteroides_stercoris | |  |
| Chen, Y. et al. 2020 | >4.0 | **HPV:**Megasphaera | **LSIL:**Prevotella amnii, HSIL:Prevotella timonensis, Shuttleworthia, Streptococcaceae | | | **CC:**Bacillus, Sneathia, Acidovorax, Oceanobacillus profundus, Fusobacterium, Veillonellaceae, Anaerococcus, Porphyromonas uenonis | | | | |  | |  |
| Dareng, E. O. et al. 2016 | ─ | **HrHPV:**Bacteroidales sp, Prevotellacese, Bacteroidales, Bacteroidia, Clostridiaceae, Peptostreptococcaceae, Leptotrichiaceae, Fusobacteriales, Fusobacteria | | | | | | | | | Rhizoblales, Alphaproteobacteria, Xanthomonadales | |  |
| Di Paola, M. et al.2017 | >2.0 | **HPV:**Sneathia, Megasphaera, Pediococcus, Brevibacterium, Pseudomonas | | **PH:**Atopobium, Faecalibacterium | | | | **TH:**Albidiferax | | | | Pseudoxanthomonas, Actinomyces, Vampirovibrio, GpⅧ, Alkanindiges | |
| Fang, B. et al. 2022 | >4.0 | **HrHPV:**Actinobacteriota, Gardnerella, Bidfidobacterium, and Atopobium | | | | | | | | | Firmicutes, Lactobacillus | |  |
| Hu, J. et al. 2022 | >4.0 | **HPV:**Bifidobacteriales, Bifidobacteriaceae, Gardnerella, uncultured_bacterium_g_Gardnerella, Coriobacteriia, Coriobacteriales, Atopobium_vaginae, Atopobiaceae, Atopobium, Clostridia, Clostridiales | | | | | | | | |  | |  |
| Huang, X. et al. 2018 | >2.0 | **16SIL:**Coriobacteriales, Actinobacteria, Coriobacteriia, Atopobiaceae | | **52SIL:**Bacillus, | | | | **58SIL:**Ruminiclostridium, Aurantimonadaceae, Aureimonas | | | |  | |
| Ivanov, M. K. et al. 2023 | >2.0 | **LSIL:**Sphingorhabdus, Noviherbaspirillum, Phenylobacterium, Pseudarthrobacter | | **surgery:**Azospirillum, Varibaculum, Bosea, Actinotignum, Propionimicrobium | | | | **NILM:**Lactobacillus, Rhodopseudomonas, Serratia, Caulobacter | | **CC:**Cutibacterium, Acinetobacter, Rhodococcus, Amaricoccus, Paracoccus | |  | |
| Kang, G. U. et al. 2021 | >7.0 | **CIN:**Gardnerella | **CC:**Streptococcus, Streptococcaceae, Peptoniphilus, Staphylococcales, Finegoldia, Anaerococcus, Clostridia, Peptostreptococcales_Tissierellales, Prevotellaceae, Prevotella, Bacteroidales, Bacteroidota, Bacteroidia | | | | | | | | Lactobacillaceae, Lactobacillus, Bacilli, Lactobacillales, Firmicutes | |  |
| Kwon, M. et al. 2019 | ≥2.5 | **CIN2*3:**Lactobacillus, Staphylococcus, Candidatus, Endolissoclinum | | | | **CC:**Akaliphilus, Pseudothermotoga, Wolbachia | | | | | Pseudoalteromonas, Psychrobacter | |  |
| Lee, J. E. et al. 2013 | ─ | **HPV:S**neathiac, Fusobacteriaceaed, Fusobacteriales | HrHPV:Sneathiac, Fusobacteriaceaed, Fusobacteriales | **postmenopausal women:**Porphyromonas, Porphyromonadaceae, Streptococcus, Streptococcaceae, Anaerococcus, Finegoldia, Peptoniphilus, unclassified, Incertae_Sedis_XI, Clostridiales, unclassified, Caulobacteraceae, Caulobacterales, Bradyrhizobium, Bradyrhizobiaceae, Methylobacterium, Methylobacteriaceae, Rhizobiales, Sphingomonas, unclassified, Sphingomonadaceae, Sphingomonadales, Campylobacter Campylobacteraceae Campylobacterales | | | | | **premenopausal women:**Lactobacillus, Lactobacillaceae, Burkholderiales | | Lactobacillales | |  |
| Lin, W. et al. 2022 | >2.0 | **HPV:**Bacteroidetes, Bacteroidales, Bacteroidia, Prevotellaceae, Prevotella, Saccharofermentans, Proteobacteria, Gammaproteobacteria, Peptostreptococcus, Acidobacteria, Megasphaera, Fusobacteria, Fusobacteriales, Clostridiales_Incertae_Sedis_XI, Soeathia, Leptotrichiaceae, Coriobacteriaceae, Veilloneellaceae, Coriobacteriales, Atopobium | | | | | | | | | Firmicutes, Bacilli, Lactobacillales, Lactobacillus, Lactobgcillaceae | |  |
| Liu, H. et al. 2022 | ≥4.0 | **HPV:**Lactobacillus iners, Lactobacillus crispatus, Escherichia coli, Lactobacillus gasseri | | **CIN:**Bifidobacterium | | | | **CC:**Prevotella, Porphyromonas, Bacteroides | | | |  | |
| Liu, S. et al. 2022 | ─ | **SHPV:**Proteobacteria, Burkholderiaceae, Peptostreptococcus, uncultureed_bacterium_g_Peptostreptococuss | | **MHPV:**Rhizobiales, α-Proteobacteria | | | | | | | Rnzobiales, Aiphaproteobacteria, Burkholderla-Caballeronia-Paraburkhoideria, Shewanella, Shewaneliaceae, Shewanela_sp_FDAARGOS_354, Alahizobium-Neorhizobium-Pararhizobium-Rhizobiums | |  |
| Ma, Y. et al. 2023 | ─ | **HSIL:**Hungateiclostridiaceae, Sneathia, Leptotrichiaceae, Fastidiosipila, Clostridia, Megasphaera | | **CC:**Fannyhessea vaginae, Prevotella, Bacteroides, Finegoldia, Vibrio, Veillonella, Peptostreptococcus, Dialister, Sneathia | | | | | | |  | |  |
| Mitra, A. et al. 2015 | ─ | **LSIL:**Lactobacillus jensenii, Lactobacillus coleohominis | | **HSIL:**Peptostreptococcus anaerobis, Anaerococcus tetradius, Sneathia sanguinegens | | | | | | |  | |  |
| Mitra, A. et al. 2020 | ─ | **PH:**Megasphaera , BVAB1, Prevotella timonensis, Gardnerella vaginalis, Sneathia, Atopobium | | **TH:**Lactobacillus spp. | | | | | | |  | |  |
| Onywera, H. et al. 2019 | >2.0 | **HPV:**Alphaproteobacteria, Rickettsiales, Actinobacteria, Bifidobacteriaceae, Bifidobacteriales, Gardnerella | | **HrHPV:**Aerococcaceae, Pseudomonadaceae, Bifidobacteriaceae, Gardnerella, Sneathia, Atopobium, Aerococcus, Pseudomonas | | | | | | | Haemophilus, Phyllobacterium | |  |
| Piyathilake, C. J. et al. 2016 | ≥2.0 | **CIN1:**Ruminococcaceae, Clostridiales | | **CIN2*3:**Lactobacillaceae, Bacteroidaceae, Porphyromonadacae, Coxiellaceae, Bacteroides, Parabacteroides, Rickettsiella, RFN20 | | | | | | |  | |  |
| Ritu, W. et al. 2019 | ─ | **HPV:**Ureaplasma, Ureaplasma parvum, Fusobacterium nucleatum | | **PH:**Prevotella timonensis, Lactobacillus delbrueckii, Eubacterium_g23 HQ746544_s, Microvirga lupini, Deinococcus swuensis, HM748650_g FJ936969_s (belonging to the family Haliangiaceae), Dialister invisus, Anaerobacterium chartisolvens, Coprococcus comes | | | | | **TH:**Rhodobacterales, Rhodobacteraceae | | Deinococcus, Thermus, Deinococci, Acidobacteria Gp4, Listeriaceae, Ectothiorhodospiraceae, Coxiellaceae, Fusobacteriaceae, Faecalibacterium, Ezakiella, Brochothrix, Diplorickettsia, Fusobacterium , aecalibacterium GL538271_s, Ezakiella massiliensis, Pseudomonas aeruginosa | |  |
| Sims, T. T. et al. 2020 | ≥2 | **HPV:**Dysplasia:Lachnospira (in the Clostridia class of bacteria) | | | | | **CC:**Betaproteobacteria, Gammaproteobacteria, Burkholderiaceae, Erysiopelotrichaceae, Synergistaceae, Comamonadaceae | | | |  | |  |
| Tango, C. N. et al. 200 | >2.0 | **CC:**Saccharibacteria_TM7, Saccharimonas, Bacillales, Propionibacteriales , Streptococcus, Massilia, Ureaplasma, Staphylococcus, Fusobacterium nucleatum, Prevotella amnii, Veillonella | | | | | | | | | Gardnerella | |  |
| Tosado-Rodríguez, E. et al. 2023 | >2.0 | **HPV:**Megasphaera, Parabacteroides, Akkermansia, Bacteroides | | | | | **HSIL:**Parabacteroides, Akkermansia | | | |  | |  |
| Usyk, M. et al. 2020 | >2.0 | **PH HrHPV visit 1:**Gardnerella vaginali, PH HrHPV visit 2:Prevotella amnii , Anaerococcus prevotii | | | | | **TH HrHPV visit 1:**Lactobacillus iners | | | |  | |  |
| Wei, B. et al. 2022 | >2.0 | **LSIL:**Streptococcus , **HSIL:**Ralstonia, Anaerococcus | | | | | | | | |  | |  |
| Wei, Z. T. et al. 2021 | >2.0 | **HPV:**Mycoplasma | | | | | **CC:**Gardnerella, Atopobium, Dialister | | | | Faecalibaculum, Rhodococcus, Roseburia, Lactobacillus, Faecalibacterium, Alloprevotella | |  |
| Wu, M. et al. 2020 | >2.0 | **SIL:**Delftia | **NILM:**Peptostreptococcaceae, Pseudomonadales, Pseudomonadaceae, Pseudomonas, Bacteroidaceae, Bacteroides | | | | | | | | Lactobacillales, Sporolactobacillaceae, Sporolactobacillus | |  |
| Wu, S. et al. 2021 | >2.0 | **HPV:**Bacilli | | **LSIL:**Lactobacillus, Xanthobacter, Thermus, Flavisolibacter, Sphingopyxis, Sediminibacterium, Geobacillus, HSIL:Sneathia | | | **CC:**Prevotella, Mycoplasma, Porphyromonas, Megasphaera, Campylobacter, Dialister, Peptoniphilus, Peptostreptococcus, Anaerococcus, WAL_1855D | | | | Phycisphaerae, Phycisphaerales, Planctomycetes, Rothia | |  |
| Xia, Y. et al. 2022 | ≥3.0 | **HrHPV:**Gardnerella | | | | | **LSIL:**Shuttleworthia | | | | Lactobacillus | |  |
| Xu, X. et al. 2023 | ─ | **HRCD:**Clostridia, Oceanospirillales, Clostridiales, Halomonadaceae, Family_X, Fusobacteriaceae, Enterococcaceae, Fusobacterium, Enterococcus, Streptococcus agalactiae | | | | | | | | | Bacilli, Lactobacillales, Lactobacillaceae, Shuttleworthia, Megasphaera, Lactobacillus, L. jensenii, Pseudomonas aeruginosa BWHPSA025, Lactobacillus helveticus, Lactobacillus acidophilus. | |  |
| Yang, Q. et al. 2020 | ─ | **HPV16**:Gardnerella vaginalis, Gardnerella_sp_304, and Gardnerella_sp_2612 (Gardnerella genus), Peptostreptococcus anaerobius, Mobiluncus curtisii, Prevotella disiens, Prevotella bivia, Prevotella amnii, Prevotella corporis (Prevotella genus), Fusobacterium nucleatum | | | | | | | | | Enterococcus sp_1140_ESPC | |  |
| Zeng, M. et al. 2023 | >2.0 | **SHPV:**Klebsiella | | **twoHPV:**Parvimonas, unidentified Christensenellacea, Candidatus competibacter, unidentified Gammaproteobacteria, Terrimonas, Leisingera, Hyphomicrobium, Terrabacter, unidentified Alphaproteobacteria and Enhydrobacter | | | **MHPV:**Ferruginibacter, Haloactinopolyspora, unidentified Rhizobiaceae, Blastochloris, Vibrio, Ornithinimicrobium, Tetragenococcus, and Castellaniella | | **PH:**Erysipelotrichia, Bacteroidaceae, Erysipelotrichaceae, Helicobacteraceae, Neisseriaceae, Streptococcaceae, Erysipelotrichales, lavobacteriales, Fusobacterium, Bacteroides, Neisseria, Helicobacter | |  | |  |
| Zeng, W. et al. 2023 | ─ | **CIN1:**Sneathia, Leptptrichiaceae | | **CIN2*3:**Escherichia Shigella | | | | | P. Bacteroidota, P. proteobacteria, Corynebacterium, Bacteroides, Corynebacteriaceae, Muribaculaceae, Corynebacteriales, Clostridia | |  | |  |
| Zhang, C. et al. 2018 | >2.0 | **HPV:**Streptococcus agalactiae, Bacteroides fragilis, Pseudomonas stutzeri, and Peptostreptococcus anaerobius | | **CIN1:**Photobacterium damselae, Lactobacillus jensenii, Atopobium vaginae | | | **CIN2*3:**Lactobacillus crispatus, Streptococccus agalactiae, Bacteroides fragilis, Campylobacter ureolyticus | | | | Lactobacillus delbrueckii | |  |
| Zhang, Y. et al. 2022 | >2.0 | **HPV:**Shuttleworthia, Clostridia Lachnospiraceae, Bifidobacterium, Ralstonia pickettii, and Lactobacillus gasseri | | | | | **CIN:**Aerococcus, Coriobacteriia, Atopobium | | | | **CIN was compared with N:**Ralstonia, Bifidobacterium, Lactobacillus gasseri, Clostridia **HPV was compared with N:**Prevotella bivia , Pseudomonadales | |  |
| Zhang, Z. et al. 2021 | ─ | **cervical Other hrHPV:**Enterobacteriaceae | | | | | **CC:**Gammaproteobacteria | | | | **cervical N:**Actinomycetales, Chitinophagaceae, Sphingobacteriales, Sphingobacterila, Bacillales, Lachnospiraceae, Ruminococcus, Ruminococcaceae, Phenylobacterium, Caulobacteraceae, Caulobacterales, Bradyrhizobium, Bradyrhizoblaceae, Methylobacterium, Methylobacteriaceae, Mesorhizobium, Phyllobacteriaceae, Rhizobiales, Sphingomonas, Sphingomonadaceae, Sphingomonadaies, Aiphaproteobacteria, **vaginal N:**Lactobacillus, Lactobacillaceae, Lactobacillales, Bacilli | |  |

N, healthy control; CC, Cervical cancer; HPV, Human papillomavirus infection; CIN, cervical intraepithelial neoplasia; CIN1, low-grade cervical intraepithelial neoplasia; CIN2*3, high-grade cervical intraepithelial neoplasia; HrHPV, high-risk human papillomavirus infection; LSIL, low-grade squamous intraepithelial lesion; HSIL, high-grade squamous intraepithelial lesion. PH, persistent HPV infections; TH, transient HPV infections; SHPV, single HPV-genotype infection; MHPV, multiple HPV-genotype infection; NILM, no intra-epithelial lesion or malignancy; HRCD, HPV-related cervical disease, HPV infection, including CIN and cervical cancer.
